# Dynamic networks connect the USP14 active site region with the proteasome interaction surface

**DOI:** 10.1002/pro.70077

**Published:** 2025-03-17

**Authors:** Johannes Salomonsson, Linda Sjöstrand, Arvid Eskilson, Dean Derbyshire, Pádraig D'Arcy, Maria Sunnerhagen, Alexandra Ahlner

**Affiliations:** ^1^ Department of Physics Chemistry and Biology, Linköping University Linköping Sweden; ^2^ Department of Biomedical and Clinical Sciences Linköping University Linköping Sweden

**Keywords:** allostery, deubiquitinases (DUBs), NMR spectroscopy, protein dynamics, ubiquitin specific proteases (USP), ubiquitin‐proteasome system (UPS)

## Abstract

Ubiquitin‐specific protease 14 (USP14) is a member of the USP family responsible for the catalytic removal of ubiquitin (Ub) from proteins directed to the proteasome, implicated in the pathogenesis of neurodegeneration and cancer. Crystallography and cryo‐EM analysis have identified loop regions crucial for the deubiquitinase activity of USP14, specifically those involved in Ub and proteasome binding. However, the structural changes in USP14 upon ligand binding to these regions are minimal, indicating significant yet uncharacterized dynamic contributions to its function. In this study, through structural and dynamical NMR experiments and functional evaluation, we demonstrate that small mutations designed to impact Ub binding and catalytic activity without disturbing the USP structure display both local and long‐range effects. The affected residues connect the catalytic site and the Ub binding region with the proteasome interaction surface through a network of loops, which show varied dynamics on the ps–ms time scale. Collectively, our findings experimentally reveal different aspects of dynamic connections within USP14, suggesting the presence of allosteric networks that link enzyme activity with regulatory function. The identification of coupled clusters of possible allostery participants in the free USP domain provides new insights into the dynamic regulation of USP14, with potential implications for understanding its role in cellular processes.

## 1 INTRODUCTION

The ubiquitin‐proteasome system (UPS) plays a crucial role in maintaining protein homeostasis by coordinating the degradation of damaged, misfolded, or short‐lived proteins. At its core is the proteasome, a large multisubunit proteolytic complex functioning as the cell's molecular shredder, and ubiquitin (Ub)—a highly conserved 8 kDa protein serving as a specific degradation tag. Ubiquitination, involving E1, E2, and E3 ligases, sequentially attaches Ub to target proteins, forming mono‐ or polyubiquitin chains. Conversely, deubiquitinases (DUBs) counter this by removing Ub from target substrates (Nandi et al., 2006; Varshavsky, 2005). Humans have approximately 100 DUBs spanning seven families, categorized by sequence and structural homology. The largest and most diverse is the ubiquitin‐specific protease (USP) family, with over 50 members, all sharing a conserved USP domain responsible for Ub binding and catalytic activity (Komander et al., 2009; Snyder & Silva, 2021).

USP14, one of three proteasome‐associated DUBs, regulates protein degradation by deubiquitinating proteasome‐bound substrates (Lee et al., 2016) and modulates proteasome activity through allosteric mechanisms (Kim & Goldberg, 2017; Peth et al., 2009). Although USP14 exhibits limited DUB activity in isolation, its catalytic efficiency is enhanced upon association with the proteasome (Lee et al., 2010; Xu et al., 2015). USP14 comprises two structured domains connected by a flexible linker: a ubiquitin‐like (Ubl) domain and a USP domain, which houses its catalytic activity. The USP14 USP domain (USP14_USP_) features a conserved open hand‐like fold, with the protease active site region situated in a groove between the “thumb” and “palm”. Upon binding, the Ub moiety is positioned in the “palm” supported by the Ub‐binding loops within the USP fold (Hu et al., 2005; Komander et al., 2009). USP14_USP_ has been characterized structurally by crystallography in its free state (PDBID: 2AYN), when bound to Ub (PDBID: 2AYO) (Hu et al., 2005), when inhibited by small molecules (PDBID: 6IIK, ‐N, ‐L, ‐M) (Wang et al., 2018), and as a catalytically inactive USP14‐C114S (PDBID: 6LVS) (Lin et al., 2020). The core structure of the USP14_USP_ (Hu et al., 2005) remains well resolved and highly consistent across USP14 structures. This includes the catalytic triad (C114, H435, D451), where USP14 appears to be properly aligned for catalysis both in the presence and absence of Ub or proteasome (Hu et al., 2005; Wang et al., 2018; Zhang et al., 2022). Interestingly, this contrasts with inactive “apo” structures of closely related USP7 and USP15, where the catalytic triad is misaligned (Hu et al., 2002; Molland et al., 2014; Ward et al., 2018). The prevailing hypothesis for USP14 autoinhibition is based on its 3.5 Å apo crystal structure, where surface so‐called blocking loops named BL1 (residues 330–342), BL2 (428–434) and BL3 (468–473) partially occupy the active site groove, presumably blocking access to the catalytic site (PDB‐ID: 2AYO; Hu et al., 2005). The enzymatic activity of free USP14 is significantly enhanced by phosphorylation of S432 in BL2, which is replicated by the phosphomimicking amino acid variant USP14‐S432E, suggesting a charge‐induced BL2 loop opening to relieve autoinhibition (Xu et al., 2015); however, no structures have confirmed this mechanism.

Recent studies have provided novel insights into USP14 as a multistate allosteric regulator on binding to the proteasome. Structurally, the Ubl domain of USP14 (USP14_Ubl_) anchors to the proteasome PSMD2 subunit (also known as Rpn1 in yeast), while its catalytic USP domain interacts with the PSMC1 (Rpt2) and PSMC2 (Rpt1) subunits (Lee et al., 2010; Lee et al., 2016). Cryo‐EM studies reveal variations in USP14's interaction with the proteasome, suggesting a model for how USP14 allosterically regulates proteasomal interdomain motion and thereby its activity (Zhang et al., 2022). In these structures, USP14 interacts with the proteasome subunits PSMC2‐AAA and PSMC1‐OB domains through a series of surface loops, many of which are unresolved in crystal structures (Aufderheide et al., 2015; Hung et al., 2022; Zhang et al., 2022). The most extensive USP14 interaction region is observed between the proteasome PSMC2‐AAA subunit and USP14 residues 371–391, a region disordered in crystal structures (Hu et al., 2005; Wang et al., 2018), and the USP14 PKL loop (residues 278–285) (Hung et al., 2022; Zhang et al., 2022). The PSMC1‐OB unit also forms sparsely distributed contact points with USP14 at BL1, BL2, and BL3 (Zhang et al., 2022). However, no direct contacts were identified between proteasome subunits and the catalytic site residues of USP14, suggesting that the activation of its proteolytic activity by the proteasome is allosterically mediated.

To fully understand proteasomal activation, there is a critical need to understand the dynamic properties of the USP14 network of functional loops in solution (BL1, BL2, BL3, PKL, and SL; residues 188–199). High‐resolution structural methods such as crystallography and cryo‐EM are limited in characterizing dynamic properties and provide little or no information for highly flexible regions, requiring alternative approaches (Corbella et al., 2023; Papaleo et al., 2016). In solution, the NMR chemical shift is highly sensitive to the local chemical environment and thereby reports on protein folding, ligand binding, dynamics, and structural populations (Skeens & Lisi, 2023; Williamson, 2013). By analysis of chemical shift perturbations and resonance intensity analyses in response to mutations, ligands, or protein activation/inhibition, NMR has evolved into a premier method to investigate allosteric networks, where thermodynamically and functionally coupled regions are reciprocally sensitive to structural or dynamic perturbations (Papaleo et al., 2016; Selvaratnam et al., 2011; Skeens & Lisi, 2023; Xu et al., 2019). Furthermore, ps–ns and μs–ms dynamics can be analyzed by NMR relaxation experiments in solution at both backbone amide and methyl groups, thereby exploring dynamically modulated function (Henzler‐Wildman et al., 2007; Manley & Loria, 2012; Xie et al., 2020). By these approaches, in‐depth experimental analysis of smaller proteins has revealed the pronounced role of dynamics in messaging through allosteric networks (Ashkinadze et al., 2022; Corbella et al., 2023; Papaleo et al., 2016; Petit et al., 2009; Tzeng & Kalodimos, 2012; Xie et al., 2020). However, due to the size of the USP domain (>40 kDa), NMR studies to date have only been pursued for USP7, focusing on ligand and inhibitor binding sites (Lello et al., 2017; Valles et al., 2022). Regarding USP14, assaying proteasome binding in solution by NMR is extremely challenging due to the high molecular weight of the system and has only been done for thermostable homologs to the catalytic 20S particle (Sprangers & Kay, 2007). Consequently, the dynamic and allosteric properties of USP domains in solution, including USP14, are as yet poorly understood.

To investigate the structure and dynamics of USP14_USP_ in solution, we have assigned the NMR resonances of the USP14_USP_ backbone and performed relaxation analysis of its fast dynamical properties. We show that in the free state, the dynamics of USP14 are observed at a wide range of time scales, from ps to ms. Importantly, loops BL1 and BL2 decorating the active site entry groove are predominantly dynamic in solution in the free state. To probe whether communicating networks of residues are already present in the free state of USP14, we compared the NMR chemical shifts of wild‐type (WT) USP14_USP_ with those of USP14_USP_ variants with single amino acid replacements inhibiting (USP14‐C114A) or activating (USP14‐S432E) USP14 catalytic function or localized in the USP14_USP_ Ub interaction surface. Through chemical shift perturbation (CSP) analysis, we observed that functional mutations collectively impact a broader region of USP14. This region is notably conserved in USP14 and partially overlaps with the proteasome binding interface. Our work provides an extended view of different aspects of dynamics in USP14 and provides experimental evidence to support the presence of allosteric regulatory networks that connect the catalytic site and Ub binding residues with the proteasome binding interface, thereby increasing the potential to target allosteric sites for USP14 inhibition.

## 2 RESULTS

### 2.1 First NMR analysis of USP14_USP_ resolves dynamic loops and transient secondary structures

**FIGURE 1:**
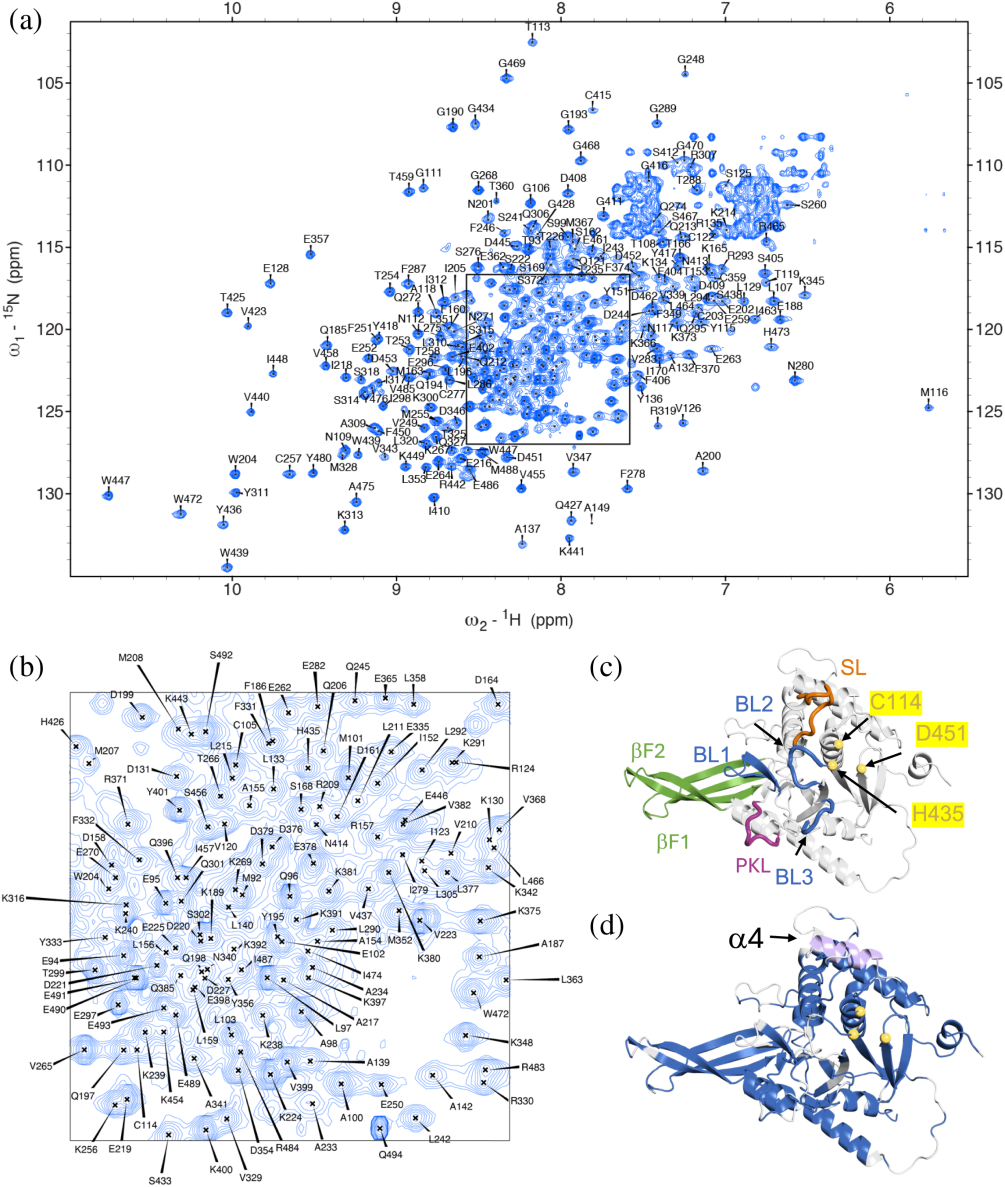
USP14 assignment, completeness and loop arrangement. (a) ^1^H–^15^N TROSY‐HSQC of ^2^H–^13^C–^15^N‐labeled USP14_91–494_ with assignments. (b) Magnification of the overlapped region indicated in (a) with corresponding assignments. (c) Model of USP14 core domain (from AF‐P54578) with functionally relevant loops highlighted. Switching loop (SL; residues 188–199) in orange, Proximal Knuckle Loop (PKL; residues 278–285) in purple, β‐fingers in green (βF1; residues 249–272 and βF2; residues 295–319), and blocking loops (BL1‐3) in blue (BL1; residues 330–342, BL2; residues 428–434 and BL3; residues 468–473). The CA atoms of the catalytic triad residues C114, H435 and D451are shown as yellow spheres. (d) Same model, with (in blue) residues with assigned chemical shifts for backbone amides and in gray unassigned residues, highlighting the unassigned helix α4 (light lilac) positioned on top of the catalytic triad residues, C114, H435 and D451 (yellow).

To study USP14_USP_ in solution, we optimized NMR conditions of ^2^H,^13^C,^15^N labeled USP14_91–494_ using TROSY‐based triple resonance experiments at 30°C. The intrinsic dynamics of USP14_USP_ allowed for the exchange of most of the amide protons without a denaturation/renaturation protocol, resulting in the backbone assignment of 81% of non‐proline amide groups (314 of 387), 87% of C_α_, 84% of C_β_, and 85% of C′ nuclei (Figure 1a,b). Due to incomplete spin systems or weak peak intensities in the 3D experiments, 55 peaks observed in the TROSY‐HSQC could not be confidently assigned.

To evaluate the structural completeness of the assignment, we mapped assigned residues onto the USP14 model from the AlphaFold Protein Structure Database (P54578) (Varadi et al., 2021). This model contains all USP14_USP_ residues, including loops (BL1‐3, SL and PKL) indicated to be of importance for function (Hu et al., 2005; Hung et al., 2022; Zhang et al., 2022) (Figure 1c) and previously unresolved regions in crystal (Hu et al., 2005) or cryo‐EM structures (Zhang et al., 2022). We recently validated this USP14_USP_ model as the best fit to small‐angle X‐ray scattering (SAXS) data in solution (Salomonsson et al., 2024). Most of the unassigned residues are in surface loops or are deeply buried in the β‐sheet core (Figure 1d). Assignments in surface loops can be challenging due to intermediate exchange and severe spectral overlap, whereas the lack of assignments for some residues located in the buried β‐sheets is likely due to limited ^2^H/^1^H exchange. Interestingly, no resonances could be assigned to residues 173–183, which according to crystal structures and the AlphaFold model form a surface‐exposed α‐helix (α4) located on top of the helix harboring the catalytic cysteine (α1, C114) (Hu et al., 2005) (Figure 1d). Since such a highly exposed helix should not have experienced any limitations in ^2^H/^1^H exchange, it is reasonable to assume that amide resonances in α4 experience chemical exchange broadening due to millisecond dynamics.

**FIGURE 2:**
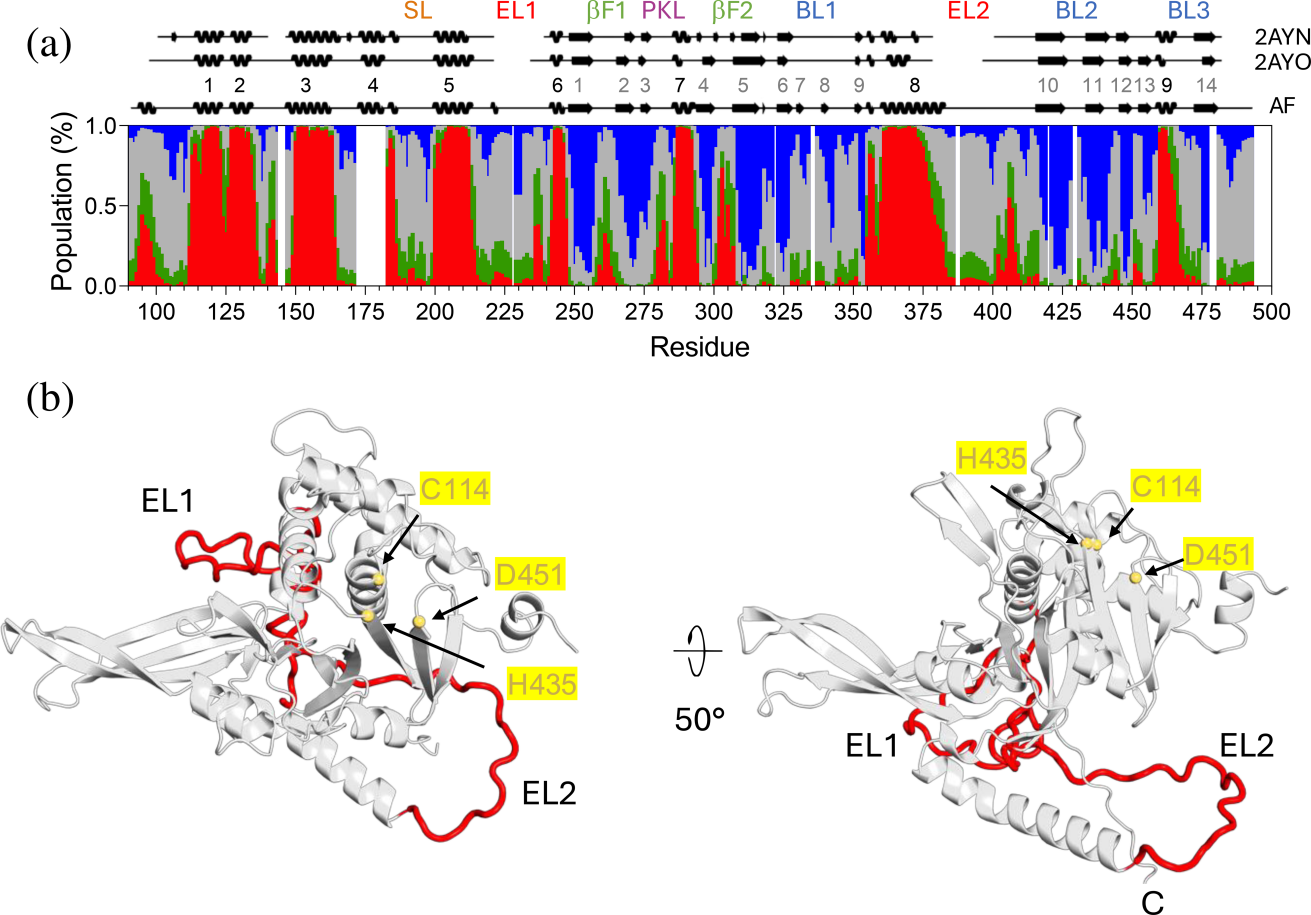
USP14 secondary structure populations. (a) Secondary structure calculation of USP14_USP_ from H_N_, N, C′, C_α_ and C_β_ chemical shifts derived by CheSPI (Nielsen & Mulder, 2021) as a function of sequence. The populations of different structure elements are colored as extended (blue), helix (red), green (turn) and coil (gray). Secondary structure elements assigned to the crystal apo and Ub bound structures PDBID: 2AYO and PDBID: 2AYN as well as the AlphaFold2 model (AF‐P54578) are indicated above the population graph, as extracted from PDBsum (Laskowski & Thornton, 2022), with secondary structure numbering indicated in black (helix) or gray (sheets) on the AF model. (b) AlphaFold model of USP14_USP_ highlighting the most flexible part of USP14_USP_ EL1 and EL2 in red, catalytic triad residues C114, H435 and D451 in yellow.

We then proceeded to assess USP14_USP_ secondary structure in solution using chemical shift secondary structure population inference (CheSPI) (Nielsen & Mulder, 2021), built on the NMR resonance assignment of H_N_, N, C′, C_α_ and C_β_ (Figures 2a and S1). Overall, the solution secondary structure compares well to that presented in the crystal structures of the USP14_USP_ in the absence and presence of Ub (PDB‐ID 2AYN, 2AYO; Hu et al., 2005), but an even better match was observed with the AlphaFold model in agreement with its excellent fit to SAXS solution data (Salomonsson et al., 2024). By the CheSPI analysis, we were able to further detail the solution populations of secondary structure in critical functional elements of USP14, including the four‐stranded β‐sheet (“fingers”) (βF1‐2) and blocking loops BL1‐BL3. In solution, βF1‐2, where Ub rests in the USP14‐Ub complex, is well defined with high β‐propensities for all four strands (β1‐β2, β4‐β5), whereas in the USP14‐apo crystal structure, the outer strands of this sheet are less distinct (β2 and β4) (Figure 2a). In solution, BL1 transiently forms a β‐finger, with maximum β‐strand populations of 20% (β7) and 60% (β8). Such β‐finger propensity is less evident in USP14_USP_ crystal structures (PDBID: 2AYO, 2AYN) but more distinct in the yeast homologue Ubp6 (PDBID: 1VJV) and clearly present when USP14 is bound to the proteasome (Zhang et al., 2022). BL2 maintains partial β propensity up until residue pair G428/G433 and may thus have some increased β‐structure propensity compared to crystal structures; lack of assignments at the tip precludes further analysis. Our data suggest that BL3 does not show regular secondary structure in solution, which agrees with the crystal structures and when USP14 is bound to the proteasome (Hu et al., 2005; Wang et al., 2018; Zhang et al., 2022).

**FIGURE 3:**
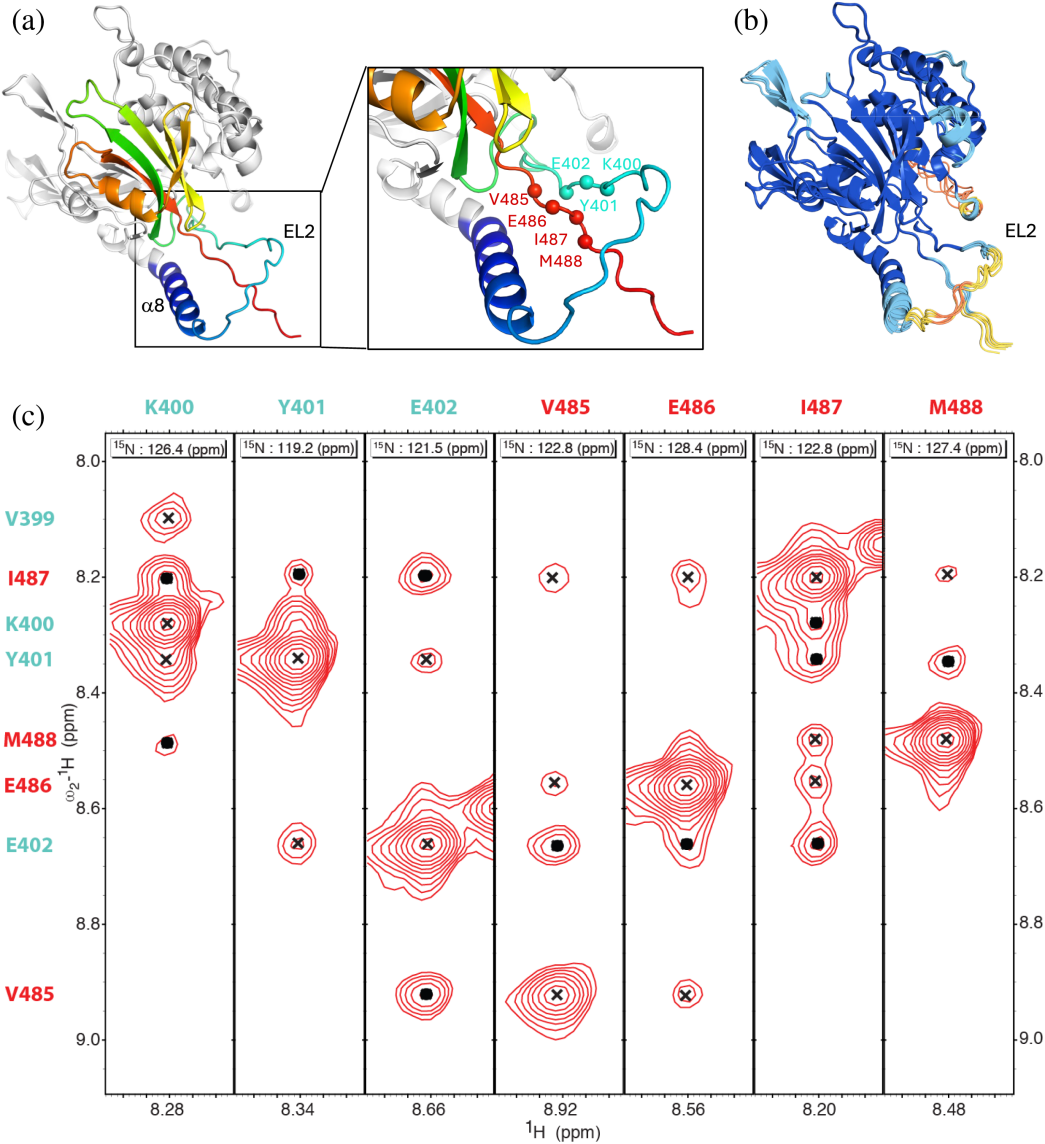
C‐terminal tail in contact with EL2. (a) AlphaFold model of USP14_USP_ from the AlphaFold Protein Structure Database (AF‐P54578) with residues 368–494 in rainbow, showing how the helix α8 (blue) ‐EL2 (cyan) motif embraces the C‐terminal tail. Residues 400–402 (cyan) and 485–488 (red) backbone amide nitrogen, showing long range NOEs in the NOESY spectra are indicated in spheres. (b) Overlay of five AlphaFold models generated with the AlphafoldServer colored according to confidence scores, Very high (dark blue, pLDDT > 90), High (blue, 90 > pLDDT > 70), Low (yellow, 70 > pLDDT > 50) and Very low (orange, pLDDT < 50). (c) Strip plots for ^15^N‐edited NOESY TROSY‐HSQC for residues 400–402 (cyan) and 485–488 (red), indicating internal/short (cross) and long range (dot) NOE's.

By NMR, we were able to significantly contribute to the improvement of existing models of two segments of USP14 that lack electron density in crystal structures (Hu et al., 2005). Based on this analysis, we here designate the extended loop regions EL1 (residues 214–242) and EL2 (residues 384–416) (Figure 2a,b). The NMR data indicate that both EL1 and EL2 lack stable secondary structure but hold transient helical turns in residues 236–239 (EL1) and 405–407 (EL2). Residues 360–383 preceding EL2 form a single, continuous helix (α8), which is stably helical until residue 375, where the helical propensity starts to gradually decline until the EL2 loop is reached. Our NMR analysis further allowed us to experimentally assess a feature of the AlphaFold USP14 structures not present in the crystal structures, where EL2 ‘extends’ beyond helix α8 wrapping around the USP14 C‐terminus without forming a knot (Niemyska et al., 2016) (Figure 3a,b). By ^15^N‐edited NOESY TROSY‐HSQC experiments, we identified H_N_‐H_N_ NOE cross peaks between residues 400–402 on EL2 and 485–487 in the C‐terminus and could thereby verify the presence of this loop arrangement in solution (Figure 3c). Together with α8, EL2 has been demonstrated to be critical for USP14 allosteric regulation of the proteasome through interactions with the AAA domain of PSMC2 (Zhang et al., 2022).

### 2.2 NMR relaxation shows a continuous community of dynamic loops and reveals intersegmental dynamic connections

**FIGURE 4:**
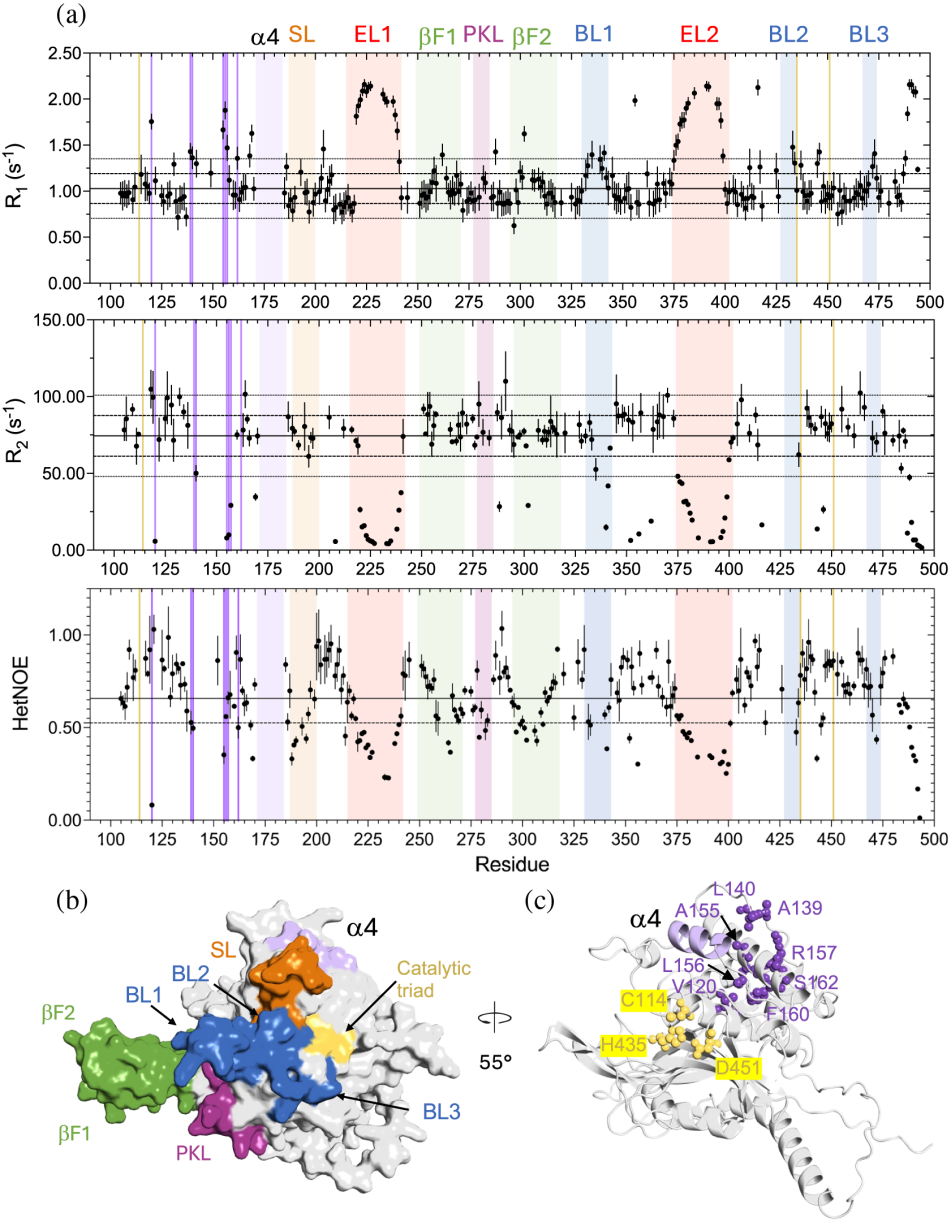
USP14 backbone NMR relaxation. Color coding throughout the figure: Helix α4 (residues 173–182) in light lilac, Switching loop (SL residues 188–199) in orange, Proximal Knuckle Loop (PKL residues 278–285) in purple, β‐fingers in green (βF1 residues 249–272 and βF2 residues 295–319), and blocking loops in blue (BL1 residues 330–342, BL2 residues 428–434 BL3 residues 468–473), extended loops in red(EL1 residue 214–242 and EL2 384–416), dynamic cluster in dark lilac, catalytic triad (C114, H435, D451) in yellow. (a) NMR relaxation data (R_1_, R_2_ and hetNOE at 900 MHz) for USP14_99–494_, revealing dynamic properties in the ps–ns range, as a function of sequence. For R_1_, R_2_ and hetNOE, the trimmed mean (see Section 4 for details) value is indicated with a straight line. The values for one (dashed) or two (dotted) standard deviations above or below the trimmed mean are indicated as lines. (b) Surface representation showing loop segments with hetNOE values below 0.5, but not as extreme R_1_ and R_2_ values as EL1 and EL2. (c) A cluster of dynamic outlier residues (lilac), form an intersegmental connection between helix α4 and helix α1 containing the catalytic C114.

To extend our understanding of internal motions in USP14, we evaluated backbone ps–ns dynamics in USP14_USP_ (Figures 4a and S2). NMR relaxation properties were determined using ^1^H–^15^N TROSY‐HSQC based longitudinal relaxation rate (R_1_) and transverse relaxation rate (R_2_) (800 and 900 MHz) as well as steady state heteronuclear {^1^H}–^15^N NOE (hetNOE) experiments (900 MHz) (Lakomek et al., 2012). NMR dynamics evaluation is often interpreted by the Lipari‐Szabo model‐free formalism to quantify the global rotational correlation time (τ_c_), the local order parameter (S^2^) and the R_ex_ contribution to relaxation due to slower motions, also called chemical exchange (Lipari & Szabo, 1982a; Lipari & Szabo, 1982b). However, this simplified formalism, often used for small proteins, has been shown to be overgeneralized for large proteins at higher magnetic fields (Chang et al., 2007). Since the molecular weight of USP14_USP_ (46 kDa) required higher magnetic fields for sensitivity and resolution, we therefore based our analysis on interpreting the relaxation experiments as such.

As expected, segments that follow the same dynamics as the core fold, within one standard deviation of the trimmed mean across the three relaxation experiments, are predominantly located within secondary structure elements (Figure 4). Higher R_1_s, lower R_2_s, and/or hetNOE values below 0.65, typically found in loops, indicate residues with high local atomic fluctuation and increased backbone flexibility in the ps–ns range. Large motions and slower dynamics (μs–ms) generate complicated relaxation patterns and, in many cases, severe line broadening and thereby loss of peaks. Our data reveal such more complex patterns for USP14_USP_, both in loops and in secondary structured segments as determined by crystallography (Hu et al., 2005) and predicted by Alphafold (Jumper et al., 2021) (Figure 2).

The most flexible regions in USP14_USP_ are EL1 and EL2 (Figures 4 and S2). Dynamically, EL1 starts at Glu219 and re‐joins the core fold dynamics at S241, entering a small helix that connects EL1 with βF1 in the finger region. Relaxation data support dynamic closure of the EL1 loop by a stable Ile218–Ile410 interaction, which is structurally observed in both crystal structures and the AlphaFold model (Varadi et al., 2021). EL2 is part of the loop arrangement that wraps around the USP C‐terminus (Figure 3a), with a progressively flexible helix (α8) that continues into the highly flexible EL2 and then anchors to the C‐terminus at residues 400–402/485–488, as shown by distinctly reduced dynamics in both 400–402 and 485–488. After leaving the loop, the C‐terminus is highly flexible.

When mapped onto the USP14_USP_ structure, the NMR relaxation data (Figure 4a) reveal a dynamic continuous community of loop segments with hetNOE values below 0.5, but not as extreme R_1_ and R_2_ values as EL1 and EL2. This community comprises the SL and structural elements anchoring onto the “catalytical” C114‐containing helix, reaches out to the tip of the β‐fingers, and includes the well‐known BL1‐BL3 and PKL loops (Figure 4b). Within this community, the relaxation experiments suggest several different regimes of flexibility. The EL1 and EL2 dynamic loops are not part of this continuous community, as they extend to the other side of the USP core fold. SL and PKL do not show a clear trend of higher or lower R_1_ or R_2_ values, suggesting significantly lower flexibility than the EL loops. Whereas BL1 shows rapid (ps‐us) dynamics by higher R_1_s together with lower R_2_s and hetNOE values, the exchange broadening due to intermediate exchange in the tip of BL2, as mentioned earlier, indicates slower dynamics (μs–ms) than in the BL1 loop, but still significantly more flexibility than in regions with defined secondary structure. The R_1_ values are increased at the c‐terminal part of BL3 and at the tips of βF1 and βF2, but no increase in R_2_ can be detected, indicating increased backbone mobility but not as much flexibility as in EL1 and EL2.

In all three NMR experiments collected at 900 MHz, we observe cases of divergent dynamics for single residues in otherwise dynamically homogeneous segments, with relaxation values distinct from the core properties in at least two of the NMR relaxation experiments. The same residues were also observed to be dynamic outliers in the 800 MHz R_1_ and R_2_ data sets. Interestingly, such dynamic outlier residues are often close to other flexible residues or segments (Y356/G416, T288/BL3) or to prolines, suggesting dynamic connections. The most extensive cluster of dynamic outlier residues includes V120 (α1), A139 & L140 (surface loop), and A155, L156, R157, F160, and S162 (α3) (Figure 4c). Here, the corresponding side chains form an intersegmental connection extending from the surface and into the helix α1 that harbors the catalytic cysteine C114 and contacts the catalytic H435 and D451. Notably, α4, which is presumably in intermediate chemical exchange (Figure 2), could be part of this dynamic cluster, being sandwiched between the intersegmental connection and the flexible SL loop (Figure 4c).

### 2.3 Single point mutations alter DUB activity with maintained structure and stability

Given that several of the identified dynamic regions in USP14 include residues that have been proposed as essential for USP14 function, we wanted to investigate whether these regions could sense any disturbances in the catalytic properties or substrate/Ub binding states of USP14. Proteasome binding is required to fully activate USP14 and has been suggested to involve a two‐state loop model for BL1 and BL2 (Hu et al., 2005). However, based on our NMR analysis and the dynamics present in these loops, the activation of USP14 cannot be explained by such a simple model. Assaying proteasome binding to USP14 in solution by NMR is prohibited by the high molecular weight of such a system (Sprangers & Kay, 2007). To resolve this, we set out to assay whether communicating networks of residues are present already in free USP14 by analyzing the effects of amino acid perturbations aimed to affect its catalytic properties or substrate binding.

We first designed and functionally characterized a set of conservative, or soft, amino acid replacements with the aim of identifying sequence positions that by themselves affect USP14 function but not its structure, so as not to confuse altered dynamics with structural changes. Based on structural analysis, we identified residues in the USP14_USP_ that interact with Ub (PDBID: 2AYO) and designed amino acid replacements aimed at reducing Ub binding at surface‐exposed positions, resulting in the designed proteins USP14‐D199A, ‐E202K, ‐Y333V, ‐S431A, ‐F331V, ‐Y436F, ‐D199A + S431A, and ‐F331V + Y333V. In the BL2 loop, we designed USP14‐S432E to mimic previously assessed activating phosphorylation at this position (Xu et al., 2015). For the catalytic site, we selected the non‐catalytic USP14‐C114A (Lee et al., 2010). All constructs were cloned, expressed, and purified as full‐length USP14 and in the same USP14_USP_ construct as used in the NMR relaxation analysis. We then assessed how these replacements affected the DUB activity, overall structure, and thermal stability of USP14_USP_ in vitro.

**FIGURE 5:**
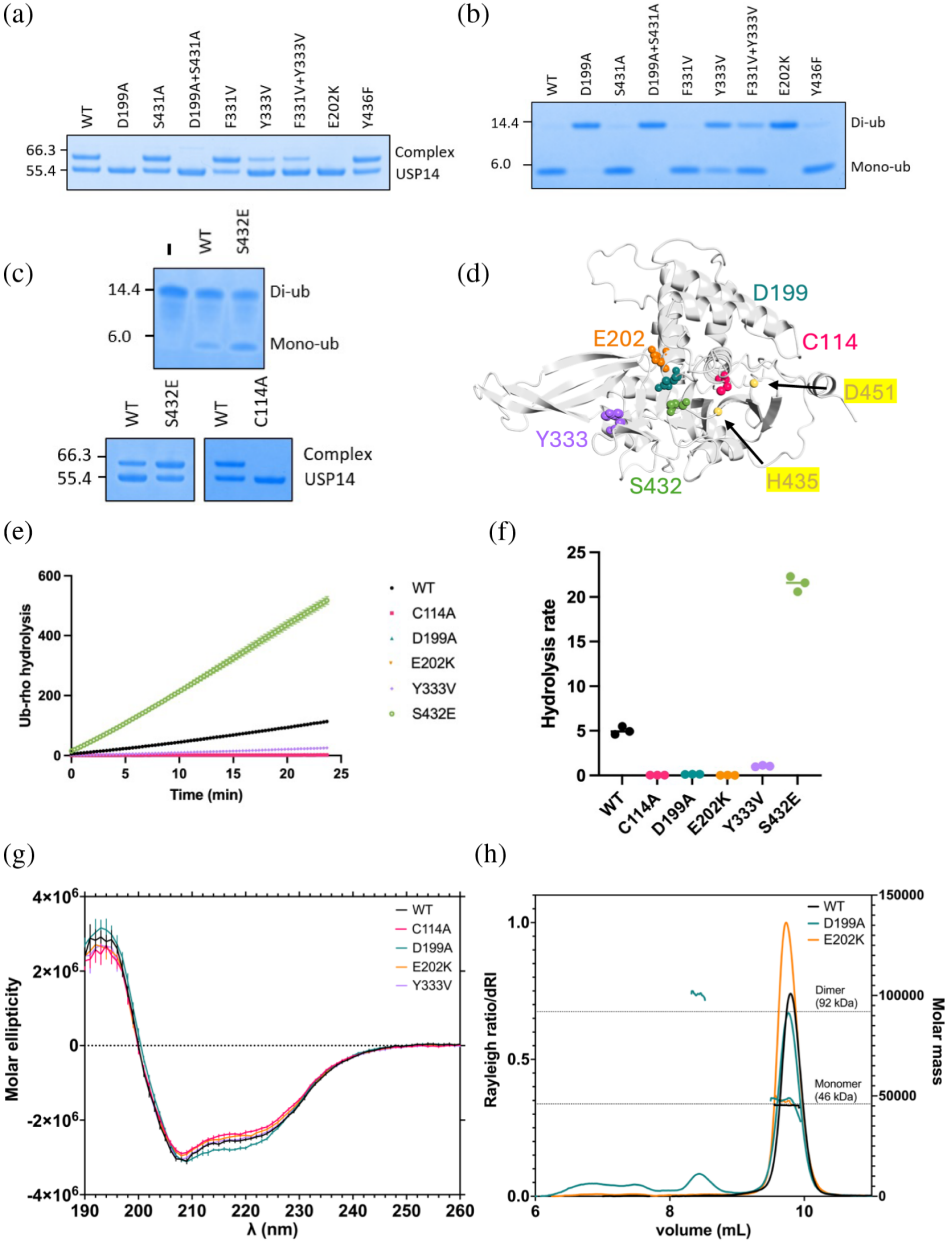
Evaluation of USP14 designed variant activity. Coloring throughout the figure USP14‐C114A (magenta), ‐D199A (teal), ‐E202K (orange), ‐Y333V (purple) and ‐S432E (green). (a) Ub‐binding gel shift assay using Ub‐propargylamide (8.5 kDa). Binding of Ub to the pocket positions the propargylamide near the active cysteine, resulting in a covalent USP14‐Ub complex with increased molecular weight. (b) USP14 was incubated with K48‐linked di‐Ub for 24 h, resulting in its cleavage into mono‐Ub. (c) Ub‐cleaving assay (upper panel) for USP14‐S432E following 6 h incubation and Ub‐binding assay (lower panel) comparing USP14‐S432E and ‐C114A. (d) Positioning of replaced amino acids shown on AlphaFold model. Catalytic residues H435 and D451 is shown in yellow for reference. (e) Representative results from Ub‐rhodamine hydrolysis assay. (f) Ub‐rhodamine hydrolysis reaction rates from three separate experiments. (g) Global secondary structure investigation using Circular dichroism on USP14‐WT, ‐C114A, ‐D199A, ‐E202K and ‐Y333V. (h) Oligomeric state detection using size exclusion chromatography multi angle light scattering (SEC‐MALS) data on USP14‐WT, ‐D199A and ‐E202K.

The activity of the full‐length designed variants was evaluated in two gel shift assays, one for binding and one for cleaving Ub with wildtype (WT) USP14 as a positive control (Figure 5a‐c) (Wang et al., 2018; Xu et al., 2015). The Ub‐binding assay utilized the Ub‐propargylamide probe. Upon Ub binding, the propargylamide moiety near the active cysteine forms a covalent bond (Figure 5a). In the cleaving assay, USP14 was incubated with K48‐linked di‐Ub, resulting in mono‐Ub by active USP14 (Figure 5b,c). USP14‐D199A and ‐E202K eliminate activity in both the binding and cleaving assays, whereas USP14‐Y333V reduced activity compared to USP14‐WT. To quantitatively compare deubiquitinating activity between USP14‐WT and USP14‐D199A, ‐E202K, ‐Y333V, ‐C114A, and ‐S432E (amino acid positions shown in Figure 5d), we conducted a Ub‐rhodamine hydrolysis assay (Figure 5e,f). Consistent with the gel shift assays, USP14‐E202K and ‐D199A exhibited no hydrolysis activity, like the non‐catalytic USP14‐C114A. On the other hand, USP14‐Y333V and ‐S432E demonstrated 20% and 430% of WT activity, respectively, providing additional evidence for the diminishing and augmenting effects of these single mutations on the DUB activity. USP14 variants with point mutations on E202 and Y333 have previously been studied and shown to partially inactivate USP14 activity (Zhang et al., 2022). As a quality control for folding and stability, the USP14_USP_ variants were analyzed with circular dichroism (CD) spectroscopy and differential scanning fluorometry (DSF) revealing highly similar secondary structure composition and stability (Figures 5g and S3).

To get first insights into the molecular effects of the mutations, ^1^H–^15^N TROSY‐HSQC spectra were recorded for ^2^H–^15^N labeled USP14_USP_‐WT and USP14‐C114A, ‐D199E, ‐E202K, ‐Y333V, and ‐S432E (Figure S4). Most of the resonances superimpose well with USP14‐WT spectra for USP14‐C114A, ‐Y333V, and ‐S432E, which, in agreement with results from CD and DSF, suggests a retained fold with only minor structural and/or dynamic changes (Figures 5g and S4). However, the USP14‐D199A spectrum is severely disturbed with an extensive loss of peaks, and the USP14‐E202K spectrum lacks 20% of the resonances identified in the USP14‐WT spectrum with 45% reduced peak intensity (Figures S4 and S5). Size exclusion chromatography coupled with multiangle light scattering (SEC‐MALS) measurements showed partial aggregation of USP14‐D199A, with slight tendencies also for USP14‐E202K at high MW (Figure 5h). Furthermore, while all designed variants show two inflection points in the first derivative of the 350/330 nm ratio, the first inflection point for USP14‐D199A significantly differs from the other designed variants (Figure S3A). To focus on local structural and dynamic effects rather than larger‐scale structural and possibly disruptive changes, we omitted USP14‐D199A and USP14‐E202K from further analysis.

### 2.4 A functionally sensitive community of residues connects the USP14 catalytic site with the proteasome binding surface

The NMR resonance chemical shift and line shape are both highly sensitive to small changes in structure and dynamics, thereby contributing a powerful tool to investigate dynamic networks in proteins (Skeens & Lisi, 2023; Williamson, 2013). The small spectral changes in the inactive USP14‐C114A and ‐Y333V and active USP14‐S432E allowed us to readily assay CSPs and relative intensities between USP14‐WT and the designed variants (Figure S5) by direct comparison of TROSY‐HSQC spectra (Figure S4), using the NMR assignment of USP14‐WT.

**FIGURE 6:**
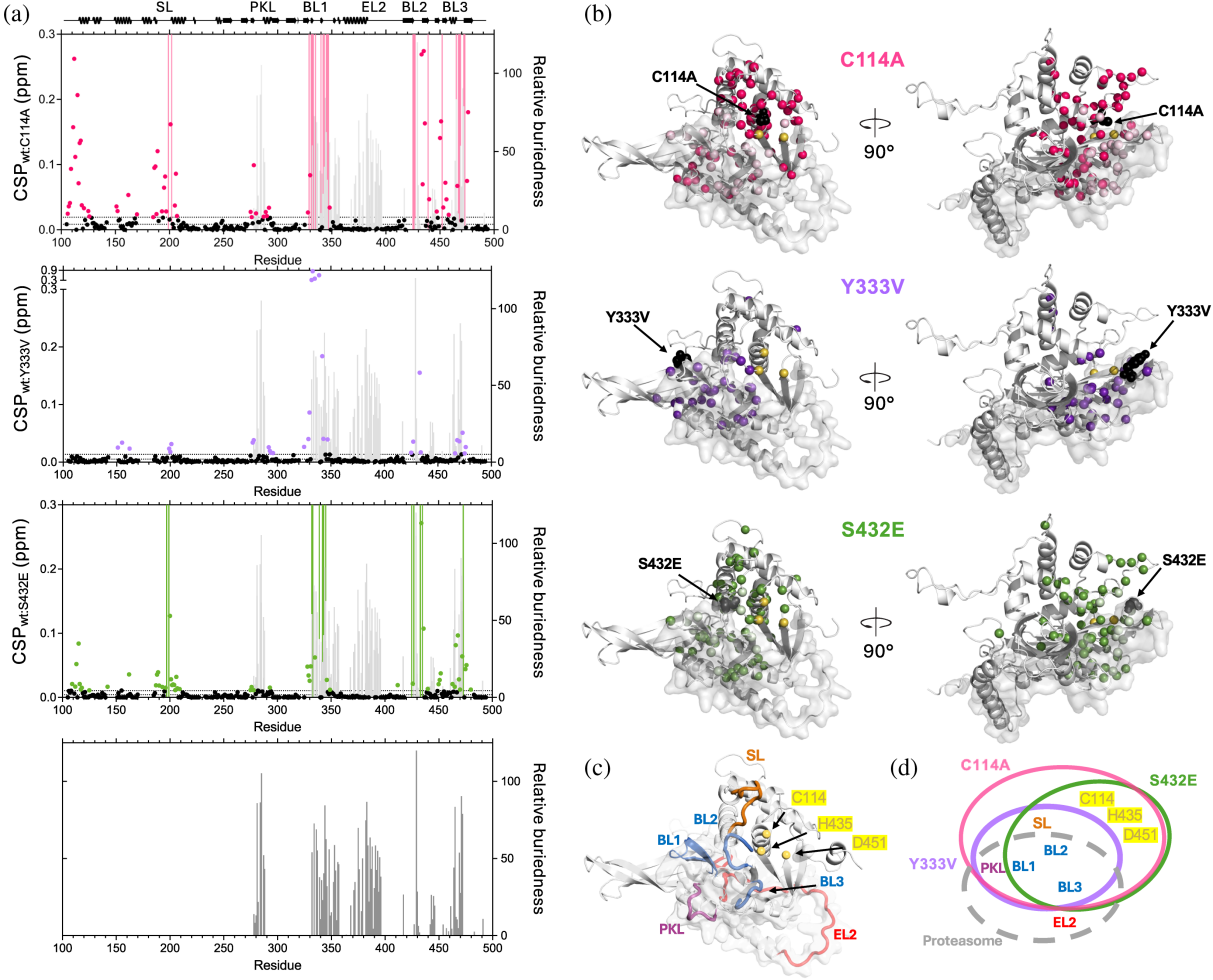
Joint and discrete USP14 variants perturbations and their connection to the proteasome interaction region. (a) CSPs between USP14 wildtype and USP14‐C114A (magenta), ‐Y333V (purple) and ‐S432E (green) are plotted as a function of sequence (dots). Missing residues are shown as colored bars. The relative buriedness (see Section 4) for all USP14 residues in all conformations identified in recent cryo‐EM USP14‐proteasome structures compared to free USP14 (Zhang et al., 2022) are shown as gray bars. For reference, the bottom panel shows only the relative buriedness. (b) Residues with significant CSPs are shown as dark magenta (USP14‐C114A), dark purple (USP14‐Y333V), or dark green (USP14‐S432E) spheres, light colored spheres indicate residues that have moved too much for correct identification or have entirely disappeared. The proteasome interface is represented in gray surface. The mutation site is shown as black spheres and the catalytic triad as yellow spheres. (c) Connected segments in USP14 as detected by CSPs include switching loop (SL, orange), Proximal Knuckle Loop (PKL, purple), Blocking Loop (BL1‐3, blue), Extended Loop 2 (EL2, red), catalytic triad (C114, H435 and D451, yellow) and proteasome interaction surface (gray, transparent). (d) Venn diagram encircling disturbed regions detected by the CSPs between USP14‐WT and USP14‐C114A (magenta), ‐Y333V (purple), ‐S432E (green).

For the catalytically inactive USP14‐C114A variant, significant CSPs are observed for 58 residues in several segments of USP14_USP_ (Figures 6, S4 and S5). In addition to expected CSPs adjacent to the mutation site, CSPs of similar magnitude are observed at positions >10 residues apart from the mutation site in the SL, PKL, BL1, BL2 (including the second catalytic residue H435) and BL3 loops, as well as in the β‐turn connecting strands β12‐β13, housing the third catalytic triad residue D451 (Figures 6 and S5). In addition, peaks corresponding to 24 residues assigned in USP14‐WT were not identified in the USP14‐C114A spectra, indicating dynamic line broadening or larger CSPs than would motivate deductive assignments (Williamson, 2013). Since only seven new peaks were detected, we assume most of the peaks that disappeared have been line broadened beyond detection, indicating a change in dynamics and/or multistate properties rather than in structural properties. For the mainly inactive USP14‐Y333V, modified in BL1, all residues could be assigned (Figure S5). Similar network effects, but less extensive and of smaller magnitude, were observed as in USP14‐C114A despite the surface‐exposed structural position of Y333. The largest CSPs were observed in the BL1 and BL2 loops, with significant CSPs also in the SL across from the Ub entry groove to the catalytic site and in the structurally more distant PKL and BL3 loops (Figure 6). Distinct from USP14‐C114A, no significant CSPs were observed for the catalytic site residues C114, H435, and D451 (Figure 6).

Interestingly, the activity enhancing USP14‐S432E (Xu et al., 2015) affects very similar loops and regions of USP14 as the inactive USP14‐C114A and partly inactive USP14‐Y333V. These include BL1, BL2, BL3, PKL, the loop preceding helix α1, helix α1 comprising C114, but with less magnitude, and the catalytic triad (Figures 6a and S5). Interestingly, the catalytic triad residue H435, which is well‐ordered with little dynamics in the WT relaxation measurements, could not be assigned in USP14‐S432E, indicating a significant structural and/or dynamic perturbation. Previous studies have suggested that phosphorylation on S432 as well as phosphomimicking USP14‐S432E both adjust the tip of BL2 to a slightly more open configuration, which was suggested to increase access to the catalytic site and thereby increase USP14 activity (Xu et al., 2015). Our CSP analysis shows more extensive dynamic and/or structural effects than would be the result of a small loop rearrangement, and with similar effects in the catalytic triad as observed in the inactive USP14‐C114A.

To better understand the structural context of the effects observed by NMR in the functional variants, we mapped residues with CSPs and line broadening effects onto the USP14 structure (Figures 6b,c and S5). The perturbed regions in USP14‐C114A, ‐S432E, and ‐Y333V are highly similar, with all three amino acid substitutions affecting residues in the SL, BL1, BL2, and BL3, and forming interconnected clusters that extend up to 25 Å from the substitution site. Although all three residues replaced are fully (S432 and Y333) or partly (C114) surface exposed, CSPs are consistently observed also for buried residues (Figure S6A). CONSURF analysis for USP14 based on PDB structures 2AYO and 2AYN shows that this continuous, partly buried and partly surface‐exposed region is highly conserved, suggesting functional importance (Figure S6B). In contrast, other regions of USP14 remain entirely unperturbed by the modifications, including βF1–βF2, the domain core, and loops located in other parts of the protein, including EL1 and EL2.

USP14 catalytic activity is enhanced by binding to the proteasome (Hu et al., 2005; Kim & Goldberg, 2017; Koulich et al., 2008; Peth et al., 2013) and reciprocally, USP14 binding to the proteasome allosterically regulates proteasome ATPase activity (Kim & Goldberg, 2017; Peth et al., 2013). As the USP14 catalytic site is not part of the direct proteasome binding surface, we asked whether the network of USP14 residues perturbed by functional mutations in the free state overlapped with USP14 residues interacting with the proteasome in cryo‐EM structures (Zhang et al., 2022). Indeed, we found that USP14 regions affected in activated/deactivated variants overlap significantly with regions partly or fully buried on binding the proteasome (Figure 6b,d). Specifically, many residues within the BL1, BL2, and BL3 loops, which were consistently affected in USP14‐C114A, ‐S432E, and ‐Y333V but >10 Å away from the site of substitution, are partly or fully buried upon proteasome binding. The PKL loop, which is a well‐defined part of the proteasome binding surface, is disturbed in all three designed USP14 variants and in particular by USP14‐C114A in the catalytic site more than 23 Å away. Interestingly, the switching loop (SL), which is distant from the proteasome interface, is also significantly disturbed. Distinct from these effects, the EL2 loop is partly buried in several of the proteasome‐bound USP14 conformations but is not affected by the mutations affecting USP14 catalytic activity.

Taken together, the NMR analysis of USP14‐C114A, ‐S432E, and ‐Y333V suggests that large, specific, and conserved networks of interconnected residues are consistently affected by small residue substitutions affecting USP14 activity and/or substrate binding. Based on the sensitivity of the NMR chemical shift to structural and/or dynamic changes (Skeens & Lisi, 2023; Williamson, 2013), our results reveal networks of interconnected residues in USP14 that can be structurally and/or dynamically sensitized by small residue alterations that affect catalytic activity. These networks comprise a community of flexible loops in and around the active site region, intersegmental dynamic connections identified by the NMR relaxation analysis, and regions known to interact with substrate (Ub) and proteasome (Figures 2, 5, and 6). As the NMR chemical shift characteristics in response to the functional mutations agree well with the presence of functionally coupled regions (Papaleo et al., 2016; Selvaratnam et al., 2011; Skeens & Lisi, 2023; Xu et al., 2019), it is a tenable hypothesis that USP14 activation by the proteasome could be allosterically mediated through this dynamic network.

## 3 DISCUSSION

In this study, we conducted a comprehensive analysis of the catalytic USP domain of USP14 using NMR to map its dynamic and structural properties in solution. Our results consistently demonstrate the highly dynamic nature of loop communities in USP14_USP_ (BL1, BL2, BL3, SL, PKL) on the ps–ns timescale, as evidenced by NMR relaxation experiments (R_1_, R_2_, hetNOE). This suggests that the USP14 loops access a broad conformational space, encompassing both “open” and “closed” states. Moreover, we observed slower dynamics in the intermediary exchange regime, as evidenced by line broadening and signal loss, particularly at the tip of the BL2 loop and in helix α4. Further analysis revealed that regions with slower dynamic properties in USP14 consistently align with residues exhibiting faster dynamics, thereby linking motions across different regions and timescales. Finally, our NMR mapping of USP14 functional mutations highlighted a community of loops and intersegmental connections that form dynamic networks extending from the active site region and switching loop to the proteasome‐binding interface (>45 Å).

The flexibility observed in the surface loops surrounding USP14's active site region, including BL1, BL2, and SL, challenges the current two‐state rigid‐body view (Hu et al., 2002; Hu et al., 2005; Hung et al., 2022; Zhang et al., 2022) and aligns with growing evidence on structurally related USPs. In USP15 crystal structures, both open and closed states of BL1, BL2, and SL loops have been observed in the absence of Ub, suggesting inherent dynamics (Priyanka et al., 2022; Ward et al., 2018). Furthermore, although crystal structures depict free USP12 with a catalytic cleft obscured by collapsed BL1 and BL2 loops, biochemical data confirm that free USP12 is fully capable of engaging its substrate, leading the authors to suggest renaming these loops as binding loops rather than blocking loops (Li et al., 2016). Numerous studies have demonstrated how enzyme activity can be both positively and negatively regulated within conformational ensembles in loops decorating the active site region (Corbella et al., 2023; Haliloglu & Bahar, 2015; Papaleo et al., 2016). For instance, studies on triosephosphate isomerase have shown that a two‐state rigid‐body loop motion can sample multiple interconverting conformations, with energetically narrow access to the catalytically active conformation achieved only in concert with substantial internal rearrangement of adjacent loops (Liao et al., 2018). Indeed, our direct experimental observation of extensive dynamics in solution in all three so‐called “blocking” (BL) loops of free USP14_USP_ suggests access to a wide array of states in the free protein, allowing for maintained dynamics in BL2 as required for substrate access and release to/from the active site region. Although we cannot yet explain USP14 autoinhibition in molecular and dynamic detail, our work prompts further investigations into the conformational ensembles of USP14 loops and how they jointly regulate USP14 activity.

Our work reveals the possibility that pre‐existing communities of residues in USP14 could be allosterically triggered to regulate USP14 activity on proteasome binding. Supporting the existence of allosteric networks, variants designed to preserve the structure or stability of USP14 nonetheless show CSP effects extending beyond surface‐exposed loops, affecting buried residues across the USP14_USP_ (Boulton & Melacini, 2016; Manley & Loria, 2012; Skeens & Lisi, 2023). As required by thermodynamics, such allosteric networks should be reciprocally sensitive to structural or dynamic perturbations (Corbella et al., 2023; Papaleo et al., 2016; Skeens & Lisi, 2023). It is therefore plausible that this community of USP14 residues could be reciprocally triggered by proteasome binding to convey allosteric messaging toward the USP14 catalytic site, resulting in USP14 activation. Interestingly, the functional mutations that were used in this work to perturb the network do not reveal stabilization of single states; rather, the opposite, as judged by observed line broadening effects (Figure S5). Based on this, our results suggest the presence of a highly dynamic allosteric signaling path between the active site region and the proteasome binding site, including the BL1 and BL2 loops previously suggested to govern USP14 activity in an open/closed manner. This view contrasts sharply with the rigid structural shifts proposed from cryo‐EM and crystallography (Hu et al., 2005; Hung et al., 2022; Zhang et al., 2022), but aligns with the concept of triggering and triggered loops put forward for lipases and enolases, where coupled motion can contribute to long‐range modulation of functional loops (reviewed in Corbella et al., 2023; Haliloglu & Bahar, 2015; Papaleo et al., 2016).

The possibility that USP14 activity is jointly regulated by differently inducible, but partly overlapping allosteric networks installing communication between the catalytic site and the spatially distinct Ub and proteasome binding sites is supported by observations in several other USPs. In USP12, two interacting proteins activate distinct yet complementary allosteric networks: UAF1 binding allosterically affects SL, while WDR20 binding allosterically activates the USP12 active site (Li et al., 2016). In USP7, full activation of USP7 requires two intramolecular allosteric interactions: by its C‐terminal tail, which dynamically binds the SL loop (Rougé et al., 2016)—and by USP7 Ubl domains 4–5, which dynamically bind at similar USP sites as where the proteasome interacts with the USP14_USP_ (Kim et al., 2019; Rougé et al., 2016). In SAGA, full activation of Ubp8 (yeast analog to human USP22) requires two allosteric regulatory touch points: Sgf1 zinc finger contacts with the loop that houses the Ubp8 active‐site cysteine, and SAGA modules Sgf1 and Sus1 occupying the same molecular space as the proteasome‐interacting EL2 region in USP14 (Samara et al., 2010). Current experimental studies of allostery have predominantly explored smaller enzymes targeting small‐molecule conversions (Ashkinadze et al., 2022; Corbella et al., 2023; Papaleo et al., 2016; Petit et al., 2009; Tzeng & Kalodimos, 2012; Xie et al., 2020). As USP14 is a sizeable protein acting on protein substrates, with activity induced by the much larger and multiconformational proteasome assembly, multiple, interconnected allosteric networks could be an intrinsic property of this protein family.

In this work, we also identified by NMR relaxation a novel dynamic intersegmental connection reaching from residues in α3 into the buried, catalytic Cys114‐carrying α1. Furthermore, this connection lies adjacent to helix α4 in USP14, which we cannot observe due to extensive line broadening. Although this USP14 region has not previously been assigned functional relevance, the helix corresponding to helix α4 in USP12 adopts shifted positions in different crystal structures, suggesting dynamics (Li et al., 2016). We note that this region corresponds to one of the dynamic USP touchpoints of the USP14_Ubl_ in full‐length USP14 as determined through joint probing by NMR and SAXS techniques (Salomonsson et al., 2024). The possible functional relevance of this intersegmental connection will need to be further investigated.

The USP14 EL2 loop, dynamically characterized for the first time in this work, together with the flanking helix α8 has been indicated by cryo‐EM modeling to form the interaction surface with the moving RPT1 AAA domain during the proteasome catalytic cycle (Zhang et al., 2022). However, EL2 is not affected by mutations in or close to the USP14 site. The extensive EL2 flexibility, possibly fine‐tuned by the connection between EL2 and the C‐terminus, may contribute enough plasticity for USP14 to remain in contact with the AAA domain throughout the different states of proteasome catalysis, while at the same time keeping the USP‐OB interaction through BL1‐3 optimized for optimal loop position and dynamics for USP activity.

Taken together, our findings reveal various aspects of dynamic connections within USP14 that link enzyme activity with regulatory functions. These networks, which include residues in the blocking loops, are interconnected through common residues, a phenomenon known as “multistate allostery” (Xu et al., 2019). The concept that USP14_USP_ signaling networks are pre‐existing, coupled, and activated by regulatory interactions with the USP fold could be crucial for future targeted drug design.

## 4 METHODS

### 4.1 Protein production

Perdeuterated 6xHis‐tagged USP14_91–494_ and USP14_99–494_ were expressed in M9+ medium enriched with ^15^N‐ammonium and deuterated ^13^C‐glucose (USP14_91–494_ only) according to the protocol developed by Cai et al. (2016). The culture was induced by 0.5 mM IPTG and harvested after 20 h at 25°C. The pellet was resuspended in 20 mM HEPES pH 7.5, 500 mM NaCl, 10 mM imidazole, 5% glycerol, 0.5 mM TCEP, 5 units/mL recombinant DNAse I, and one EDTA‐free protease inhibitor cocktail tablet per 75 mL and lysed by cell disruption at 25 psi at 4°C. Protein was purified as previously described (Selvaraju et al., 2019) but with a Hiload 16/600 Superdex 200 pg. column (GE Healthcare) for size exclusion chromatography (SEC). No unfolding and refolding of USP14 to optimize hydrogen exchange was pursued during purification due to significantly reduced yields during refolding.

### 4.2 NMR spectroscopy

Backbone assignment data were collected on a Bruker Avance III HD 800 MHz spectrometer equipped with a 3 mm TCI cryoprobe at the Swedish NMR center. ^2^H, ^13^C, ^15^N labeled USP14_91–494_ were concentrated to 0.47 mM in 20 mM HEPES, pH 7.5, 100 mM NaCl, 0.5 mM TCEP, 0.02 mM NaN_3_, and 10% D_2_O. All spectra were processed with NMRpipe (Delaglio et al., 1995) or mddNMR (Pedersen et al., 2021). Backbone resonance assignment experiments were conducted at 30°C for USP14_91–494_ using TROSY‐based HSQC, HNCA, HNcoCA, HNCACB, HNcoCACB, HNCO, HNcaCO, ^15^N‐edited NOESY‐TROSY‐HSQC experiments, and resonances were assigned by combining FLYA (Schmidt & Güntert, 2012), COMPASS (Niklasson et al., 2015) and manual assignment in NMRFAM‐SPARKY (Lee et al., 2015). Complementary assignments for loops were achieved by combining the new experiments with spectral information from the ^13^C,^15^N labeled USP14_1–494_, where crowding was less of an issue (Salomonsson et al., 2024). CheSPI (Nielsen & Mulder, 2021) was utilized for secondary structure populations based on N, H_N_, C_α_, C_β_, and C′ chemical shifts.

TROSY‐HSQC was recorded for USP14_99–494_ variants using 32 transients at 25°C with the concentrations 0.89 (USP14‐WT), 0.53 (USP14‐C114A), 0.28 (USP14‐D199A), 0.89 (USP14‐E202K), 1.07 (USP14‐Y333V) and 0.58 (USP14‐S432E) mM.

Peak intensity analysis of USP14_99–494_ variants was performed by calculating the intensity ratio between the variants and WT after normalization for concentration. CSPs between variant and WT USP14_99–494_ were calculated according to:

$$\mathrm{CSP}=\sqrt{{\Delta \delta }_{\mathrm{HN}}^{2}+{\left(\frac{{\Delta \delta }_{N}}{R_{\text{scale}}}\right)}^{2}}$$

with the scaling factor *R*_scale_ = 6.5. The trimmed mean CSP and corresponding standard deviation (*σ*) as shown in Figure 6 were calculated stepwise by first calculating the mean and *σ* of the whole data set, after which any value exceeding one *σ* was excluded and a new mean and *σ* was calculated for the remaining values. This was done in two iterations.

### 4.3 Relaxation data collection and analysis

TROSY‐optimized ^15^N R_1_, R_2_, and hetNOE experiments (Lakomek et al., 2012) were collected as pseudo‐3D at 25°C using a Bruker Avance III HD 900 MHz spectrometer equipped with a 3 mm TCI cryoprobe at the Swedish NMR center, on USP14_99–494_ with a concentration of 0.89 mM in the same buffer as above. For the relaxation measurements, the USP construct was slightly shortened in the N‐terminus compared to the assignment experiments to exclude the flexible region 91–98 (Salomonsson et al., 2024). The R_1_ and R2 experiments were collected using 12 different relaxation delays and 2 duplicates: 0.08, 0.08, 0.16, 0.24, 0.32, 0.4, 0.4, 0.56, 0.72, 0.8, 1.04, 1.2, 1.6, and 2.4 s, and the R_2_ experiment with 10 different relaxation rates, one duplicate, and one triplicate: 0.008, 0.016, 0.016, 0.016, 0.031, 0.047, 0.063, 0.094, 0.110, 0.110, 0.125, 0.156, 0.203, and 0.250 s for R_2_. The steady‐state hetNOE values were determined from the ratios of the average intensities of the peaks with and without 5 s proton saturation.

The NMR relaxation data was analyzed using the software PINT (Ahlner et al., 2013; Niklasson et al., 2017) in which peaks were integrated, fitted with a combination of Gaussian and Lorentzian line shapes, and relaxation parameters were determined. Errors were determined from the jackknife approach for the R_1_ and R_2_ experiments, while hetNOE errors were determined based on measured background noise levels using the following relationship (Farrow et al., 1994):

$$\frac{\sigma \mathrm{NOE}}{\mathrm{NOE}}=\sqrt{{\left(\frac{\sigma I_{\mathrm{sat}}}{I_{\mathrm{sat}}}\right)}^{2}+{\left(\frac{\sigma I_{\text{unsat}}}{I_{\text{unsat}}}\right)}^{2}}$$

Residues with relaxation errors larger than 20% for the relaxation parameter were excluded from the dataset, together with residues with bad 3D fits or overlapping peaks. The trimmed mean and corresponding σ were calculated as for the CSP.

### 4.4 Structure modeling using AlphaFold server

AlphaFold3 (Abramson et al., 2024) through the AlphaFold Server was used to generate five models of USP14 using standard settings.

### 4.5 Solvent accessibility calculations

Naccess (Hubbard & Thornton, 1993) was utilized to determine solvent accessibilities for residues in the AlphaFold prediction model of apo USP14 and USP14 in complex with the proteasome (PDB‐ID 7W3A, 7W3B, 7W3C, 7W3F, 7W3G, 7W3H, 7W3I, 7W3J, 7W3K, 7W3M), using the default probe size of 1.40 Å. For USP14 in complex with the proteasome, the USP14 and Ub chains were copied to new PDB files from each cryo‐EM structure to only determine the binding interface between USP14 and the proteasome. Naccess was executed for both USP14 + Ub and the full USP14 + Ub + proteasome complex. The relative buriedness was obtained by subtracting the summed all‐atom relative solvent accessibility of USP14 + Ub + proteasome residues from USP14 + Ub. Amino acids with relative buriedness >10 were considered buried.

### 4.6 Gel shift assays

Two gel shift assays were performed to evaluate the activity of the designed USP14 binding pocket variants. To assess binding to Ub‐propargylamide (Ub‐PA), 3 μM USP14_1–494_ was incubated with 6 μM Ub‐PA (Bio‐Techne, U‐214‐050) at room temperature for 24 h in a reaction buffer containing 20 mM HEPES (pH 7.5), 150 mM NaCl, 0.5 mM TCEP, and 5% DMSO. The same assay was conducted with Ub‐vinylsulfone (Ub‐VS, Biotechne, U202‐050), yielding comparable results (data not shown). Ub‐PA was selected for further analysis due to its lower reactivity, which requires proper docking of ubiquitin into the binding pocket prior to covalent attachment.

Di‐ubiquitin cleavage activity was tested by incubating 6 μM USP14 with 11.6 μM K48‐linked di‐ubiquitin (Bio‐Techne, UC‐200B‐025) at 30°C for 24 h in a buffer containing 16 mM HEPES (pH 7.5), 120 mM NaCl, and 0.40 mM TCEP. All assays were performed at room temperature for 24 h, except for those involving the S432E phosphomimetic, which were incubated for 6 h to assess differences in ubiquitin binding and cleavage activity.

Reaction products were analyzed by SDS‐PAGE, followed by Coomassie staining. Three independent experiments were performed, and one representative gel is shown.

### 4.7 DUB activity assay

Samples of 5 μM USP14 were prepared in triplicates in a black 384‐well plate (corning 3820) with a reaction volume of 20 μL. The assay buffer contained 50 mM HEPES pH 7.5, 50 mM NaCl, 5 mM MgCl_2_, 1 mg/mL BSA, 1 mM DTT, and 2 mM ATP. 1 μM Ub‐rhodamine (Bio‐Techne, U‐555‐050) was added to start the reaction. The hydrolysis of Ub‐rhodamine was monitored with a Promega plate reader at 475 nm at 37°C. The average background was subtracted. Three separate experiments were performed; the reaction rates in each experiment were calculated by simple linear regression, and significance was evaluated by the *t*‐test in Graphpad Prism 10.

### 4.8 Circular dichroism

Circular dichroism measurements were performed on a Chirascan spectrometer (Applied Photophysics) using a quartz cuvette with a 1 mm pathlength. All samples were dialyzed against 10 mM phosphate buffer, pH 7.5, and centrifuged to remove aggregates. The wavelength scans were measured in samples containing approximately 3 μM USP14, determined by Nanodrop and theoretical extinction coefficients for each construct. CD spectra for USP14_99–494_ were recorded at 20°C in the wavelength range 190–260 nm with 1 nm steps, 1.5 seconds per point, and 10 repeats. Spectra for USP14_1–494_ were recorded with 2 nm steps. The average background of the dialysis buffer was subtracted, and the units were converted to molar ellipticity based on protein concentration measured in the sample by the DC protein assay kit (Bio‐Rad) using USP14‐WT as the standard. Spectra for USP14‐S432E were collected separately, and the units were converted based on concentrations measured by Nanodrop.

### 4.9 NanoDSF

NanoDSF was measured in the dialyzed samples prepared for CD spectroscopy. The samples were centrifuged to remove aggregates before the concentration was measured by Nanodrop (Implen) and they were then diluted to 1 mg/mL with 10 mM phosphate buffer pH 7.5. Unfolding was monitored in triplicates by Prometheus NT.48, in nanoDSF grade high‐sensitivity capillaries, with a temperature increase of 1°C/min. *T*_m_ values were calculated by the accompanying software, and standard deviations are used as an error estimate.

### 4.10 SEC‐MALS

Multi‐angle light scattering (MALS) analysis was performed following size‐exclusion chromatography (SEC) using an in‐line multi‐detector instrument from Wyatt Technologies: consisting of DynaPro Nanostar and Mini‐Dawn TREOS detectors coupled to an OptiLab T‐Rex refractometer. 20 μL of USP14_99–494_ of either USP14‐WT, ‐E202K, or ‐D199A was injected onto a Superdex 75 Increase 10/300 analytical column (GE Healthcare) equilibrated in 20 mM HEPES (pH 7.5), 150 mM NaCl, and 0.5 mM TCEP at a flow rate of 0.5 mL/min. The load concentration was 320 μM for USP14‐WT, 470 μM for ‐E202K, and 423 μM for ‐D199A. Scattering data were used in combination with concentration estimates obtained from differential refractive index measurements (using a dn/dc = 0.185 mL/g) to deduce analyte molecular weight (MW). The measurements were done at 20°C. The MW distribution of species eluting from the column was determined using ASTRA7 software (Wyatt Technology).

## AUTHOR CONTRIBUTIONS

**Johannes Salomonsson:** Conceptualization; investigation; validation; visualization; writing – original draft; formal analysis; data curation; writing – review and editing; supervision; resources. **Linda Sjöstrand:** Formal analysis; visualization; writing – review and editing; investigation; validation; resources. **Arvid Eskilson:** Data curation; formal analysis; visualization; writing – review and editing; investigation; resources. **Dean Derbyshire:** Investigation; writing – review and editing; validation; resources. **Pádraig D'Arcy:** Conceptualization; supervision; writing – review and editing; writing – original draft; validation; investigation; funding acquisition; formal analysis; project administration. **Maria Sunnerhagen:** Conceptualization; supervision; writing – original draft; writing – review and editing; funding acquisition; data curation; formal analysis; project administration; visualization. **Alexandra Ahlner:** Conceptualization; data curation; formal analysis; visualization; investigation; writing – review and editing; writing – original draft; project administration; supervision.

## Supporting information


**Figure S1.** CheSPI results Summary of CheSPI evaluations based on chemical shift data from USP14_USP_ (91–494), For reference the secondary structure elements from the Alphafold model is shown and annotated on top (A) Weighted difference between observed and predicted shifts shown with blue, red, black, cyan, and magenta dots for C′, C_α_, C_β_, H_N_, and N, respectively. (B) CheSPI components (Blue and red) and CheZOD Z‐scores (black). Green dashed lines at Z = 8.0 for reference, CheZOD Z‐scores <8 are classified as disordered. (C) Bar plot colored according to the CheSPI color scheme. CheZOD Z‐scores are used for bar heights. (D) Secondary structure populations as shown in Figure 2. (E) Illustration of the most confident secondary structure prediction. (F) A visual interpretation of the CheSPI result mapped onto the AlphaFold2 structure, extending the color scheme in C to provide an intuitive overview of the local structure and dynamics of UPS14 based on its chemical shifts (Figure S1; Nielsen & Mulder, 2021).

**Figure S2.** NMR relaxation data 800 MHz. NMR relaxation data (R_1_ and R_2_ at 800 MHz) for USP14_99–494_, revealing dynamic properties in the ps–ns range, as a function of sequence. For R_1_ and R_2_, the trimmed mean value is indicated with a straight line. The values for one (dashed) or two (dotted) standard deviations above or below the trimmed mean are indicated as lines. Color coding: Helix α4 light lilac, Switching loop (SL residues 188–199) in orange, Proximal Knuckle Loop (PKL residues 278–285) in purple, β‐fingers in green (βF1 residues 249–272 and βF2 residues 295–319), and blocking loops (BL1‐3) in blue (BL1 residues 330–342, BL2 residues 428–434 BL3 residues 468–473), Extended loops (EL1‐2) (EL1 residue 214–242 and EL2 384–416) in red, dynamic cluster in dark lilac, catalytic triad C114, H435, D451 in yellow.

**Figure S3.** Supplementary evaluation of designed variants activity. (A) First derivative of thermal stability of aromatic environment detected by differential scanning fluorescence (DSF) on USP14_99–494_ USP14‐WT (black), ‐C114A (pink), ‐D199A (teal), ‐E202K (light blue) and ‐Y333V (purple). (B) Summary of DSF results. (C) Global secondary structure investigation using Circular dichroism on USP14_1–494_‐WT (black), ‐C114A (pink), ‐D199A (teal), ‐E202K (light blue) and ‐Y333V (purple).

**Figure S4.** NMR characterization of USP14 designed variants. ^1^H–^15^N TROSY‐HSQC spectra USP14_99–494_ for designed variant (red) overlayed on wildtype (black). USP14‐WT spectrum on top left for reference.

**Figure S5.** Chemical shift and intensity perturbations of USP14 designed variants. (A) Chemical shift perturbations comparing USP14‐WT and USP14‐C114A, ‐E202K, ‐Y333V and ‐S432E plotted as a function of sequence. Red lines show missing peaks or peaks that have moved so much that assignment was not possible indicating large perturbations. (B) CSPs (pink) and missing peaks (red) from (A) are plotted on AlphaFold model. (C) Intensity ratios normalized by concentration USP14‐WT and USP14‐C114A, ‐E202K, ‐Y333V and ‐S432E. Red (see A).

**Figure S6.** Allosteric networks coincide with conservation. (A) CSPs between USP14‐WT and USP14‐C114A (magenta), ‐S432E (green) and ‐Y333V (purple) of residues that have a summed all‐atom relative solvent accessibility <10 determined by Naccess in the USP14 AlphaFold model. (B) Consurf DB (consurf.tau.ac.il) visualizations of residue conservation mapped onto the USP14_USP_ crystal structure (PDBID: 2AYN, same result for 2AYO). The color scale goes from deep purple (highly conserved) over white to dark green (not conserved).

## Data Availability

The data that support the findings of this study are openly available in Backbone ^1^H, ^13^C and ^15^N Chemical Shift Assignments and ^15^N Relaxation data at https://bmrb.io/data_library/summary/index.php?bmrbId=52667, reference number 52667.
