# Supplementary figures and images for: Dynamic networks connect the USP14 active site region with the proteasome interaction surface
Source: Protein Sci. 2025 Mar 17;34(4):e70077. doi: 10.1002/pro.70077 (PMC11912437; doi:10.1002/pro.70077)

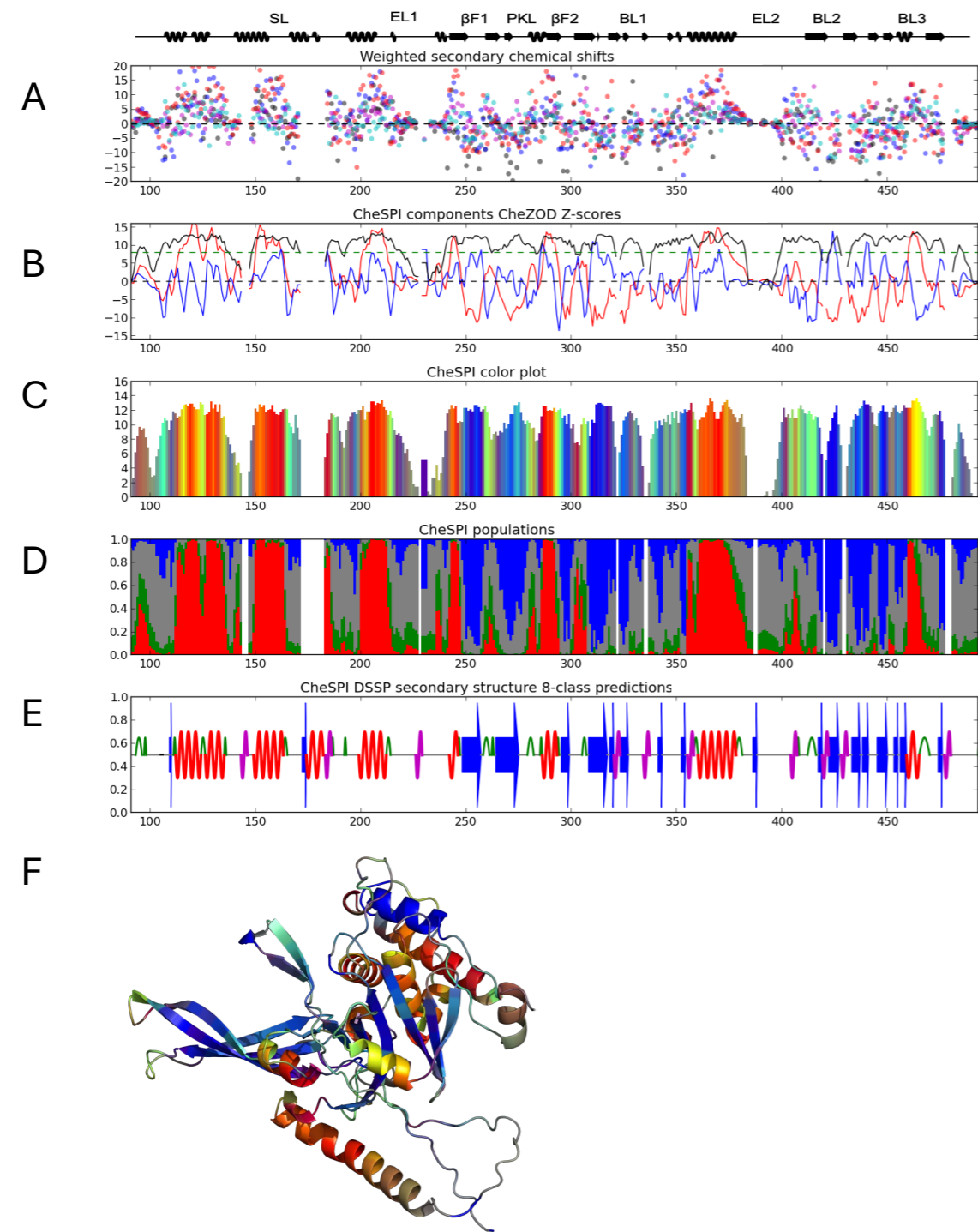

Supplement: Supplementary file 1 — Figure S1. CheSPI results Summary of CheSPI evaluations based on chemical shift data from USP14USP (91–494), For reference the secondary structure elements from the Alphafold model is shown and annotated on top (A) Weighted difference between observed and predicted shifts shown with blue, red, black, cyan, and magenta dots for C′, Cα, Cβ, HN, and N, respectively. (B) CheSPI components (Blue and red) and CheZOD Z‐scores (black). Green dashed lines at Z = 8.0 for reference, CheZOD Z‐scores <8 are classified as disordered. (C) Bar plot colored according to the CheSPI color scheme. CheZOD Z‐scores are used for bar heights. (D) Secondary structure populations as shown in Figure 2. (E) Illustration of the most confident secondary structure prediction. (F) A visual interpretation of the CheSPI result mapped onto the AlphaFold2 structure, extending the color scheme in C to provide an intuitive overview of the local structure and dynamics of UPS14 based on its chemical shifts (Figure S1; Nielsen & Mulder, 2021). [file PRO-34-e70077-s006.pdf]

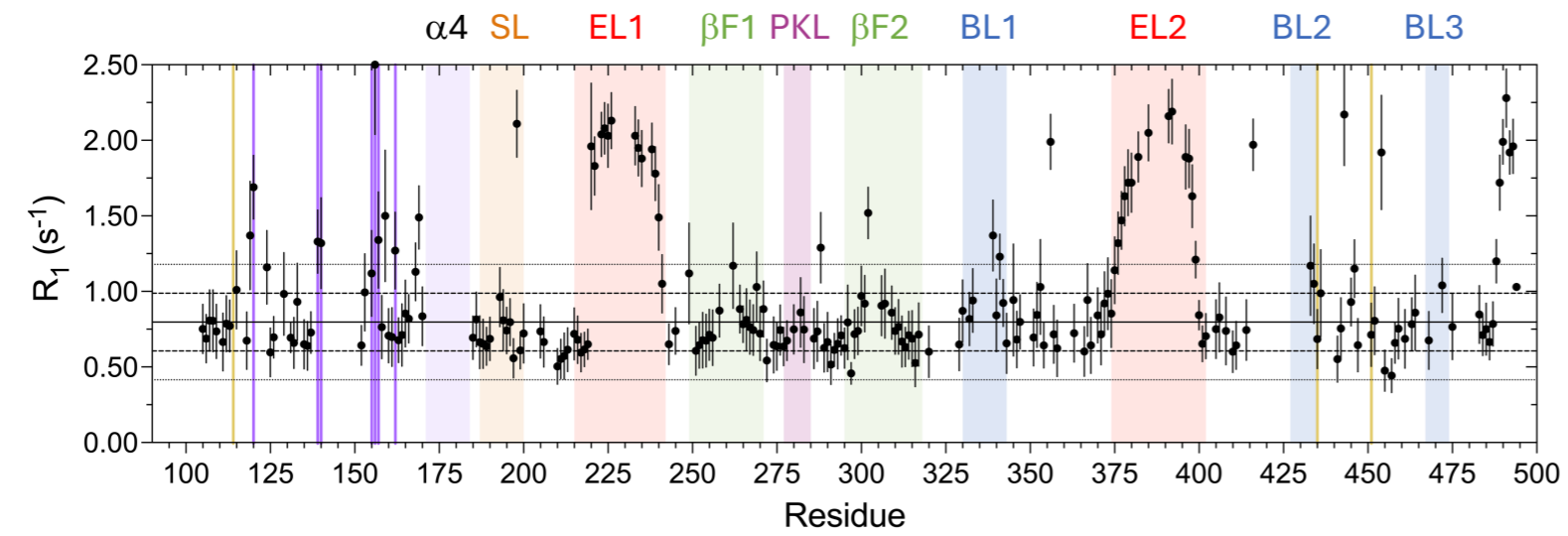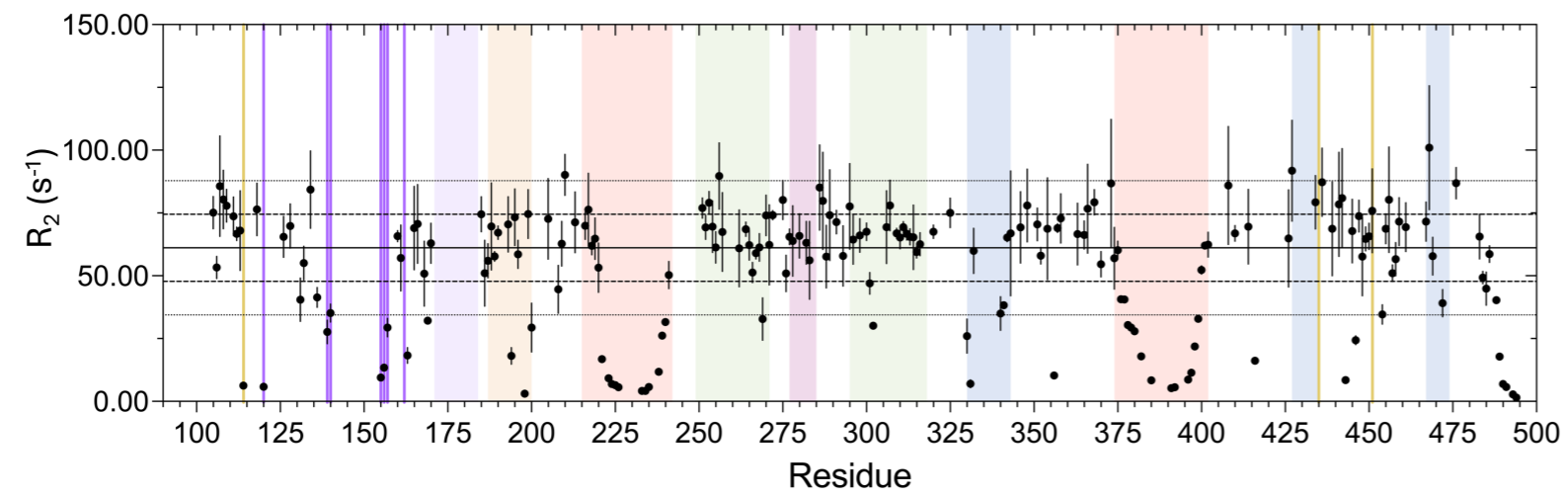

Supplement: Supplementary file 2 — Figure S2. NMR relaxation data 800 MHz. NMR relaxation data (R1 and R2 at 800 MHz) for USP1499–494, revealing dynamic properties in the ps–ns range, as a function of sequence. For R1 and R2, the trimmed mean value is indicated with a straight line. The values for one (dashed) or two (dotted) standard deviations above or below the trimmed mean are indicated as lines. Color coding: Helix α4 light lilac, Switching loop (SL residues 188–199) in orange, Proximal Knuckle Loop (PKL residues 278–285) in purple, β‐fingers in green (βF1 residues 249–272 and βF2 residues 295–319), and blocking loops (BL1‐3) in blue (BL1 residues 330–342, BL2 residues 428–434 BL3 residues 468–473), Extended loops (EL1‐2) (EL1 residue 214–242 and EL2 384–416) in red, dynamic cluster in dark lilac, catalytic triad C114, H435, D451 in yellow. [file PRO-34-e70077-s002.pdf]

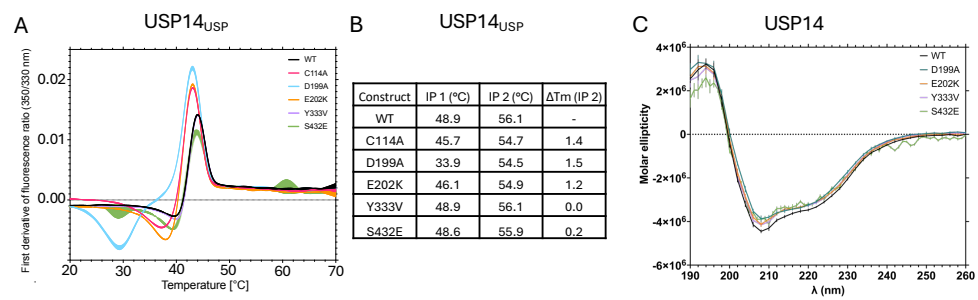

Supplement: Supplementary file 3 — Figure S3. Supplementary evaluation of designed variants activity. (A) First derivative of thermal stability of aromatic environment detected by differential scanning fluorescence (DSF) on USP1499–494 USP14‐WT (black), ‐C114A (pink), ‐D199A (teal), ‐E202K (light blue) and ‐Y333V (purple). (B) Summary of DSF results. (C) Global secondary structure investigation using Circular dichroism on USP141–494‐WT (black), ‐C114A (pink), ‐D199A (teal), ‐E202K (light blue) and ‐Y333V (purple). [file PRO-34-e70077-s001.pdf]

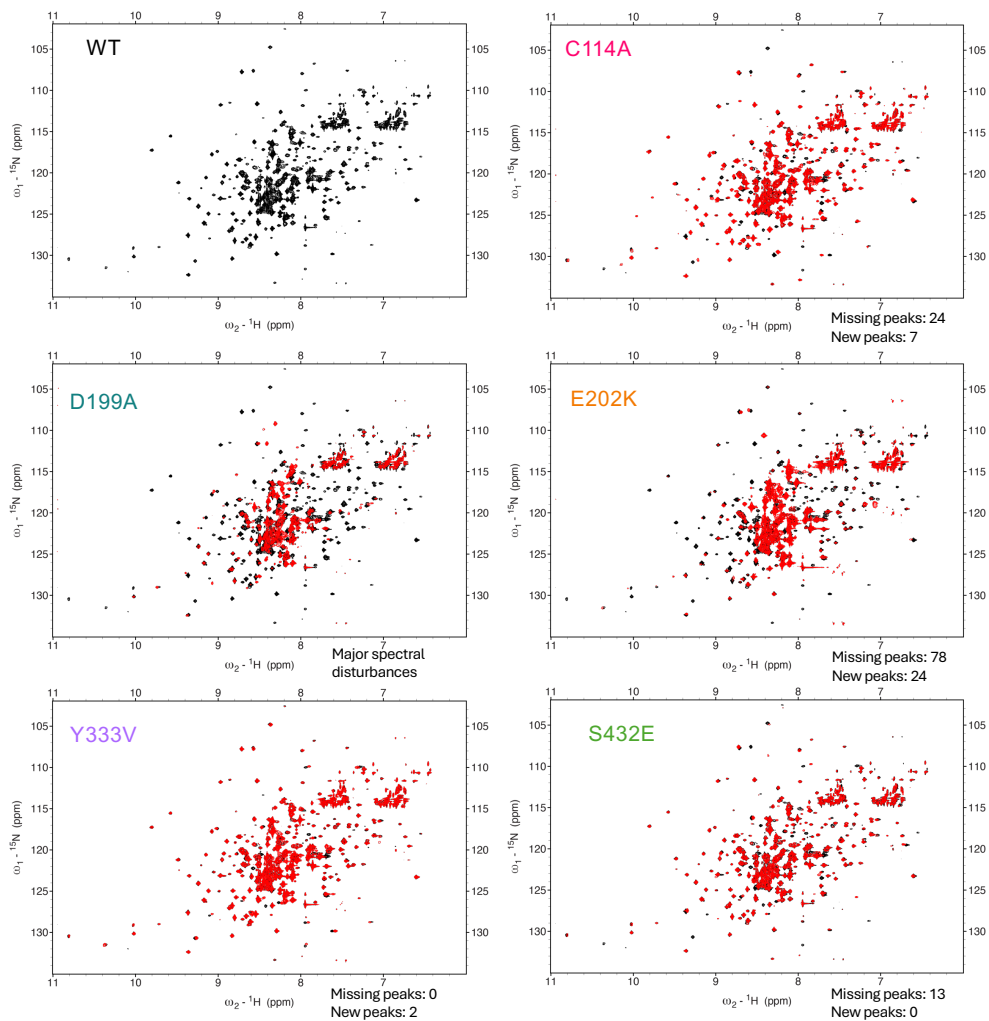

Supplement: Supplementary file 4 — Figure S4. NMR characterization of USP14 designed variants. 1H–15N TROSY‐HSQC spectra USP1499–494 for designed variant (red) overlayed on wildtype (black). USP14‐WT spectrum on top left for reference. [file PRO-34-e70077-s005.pdf]

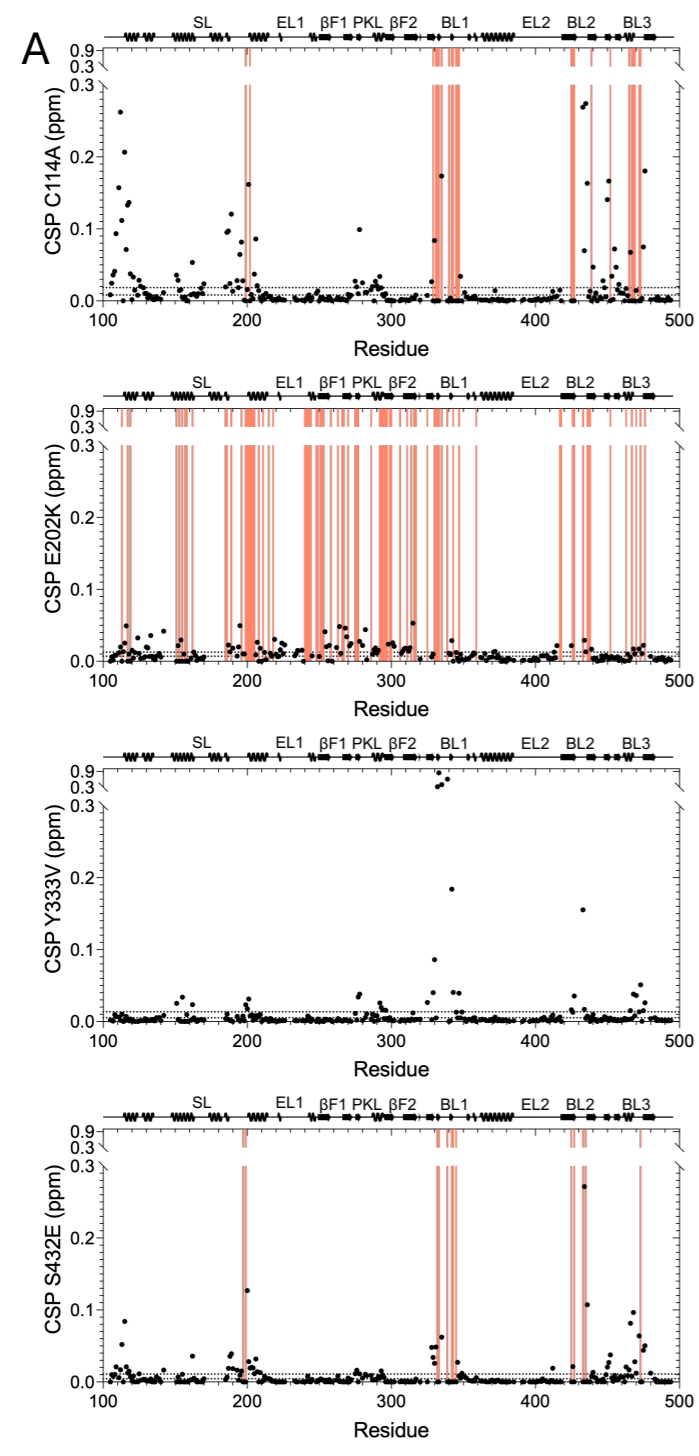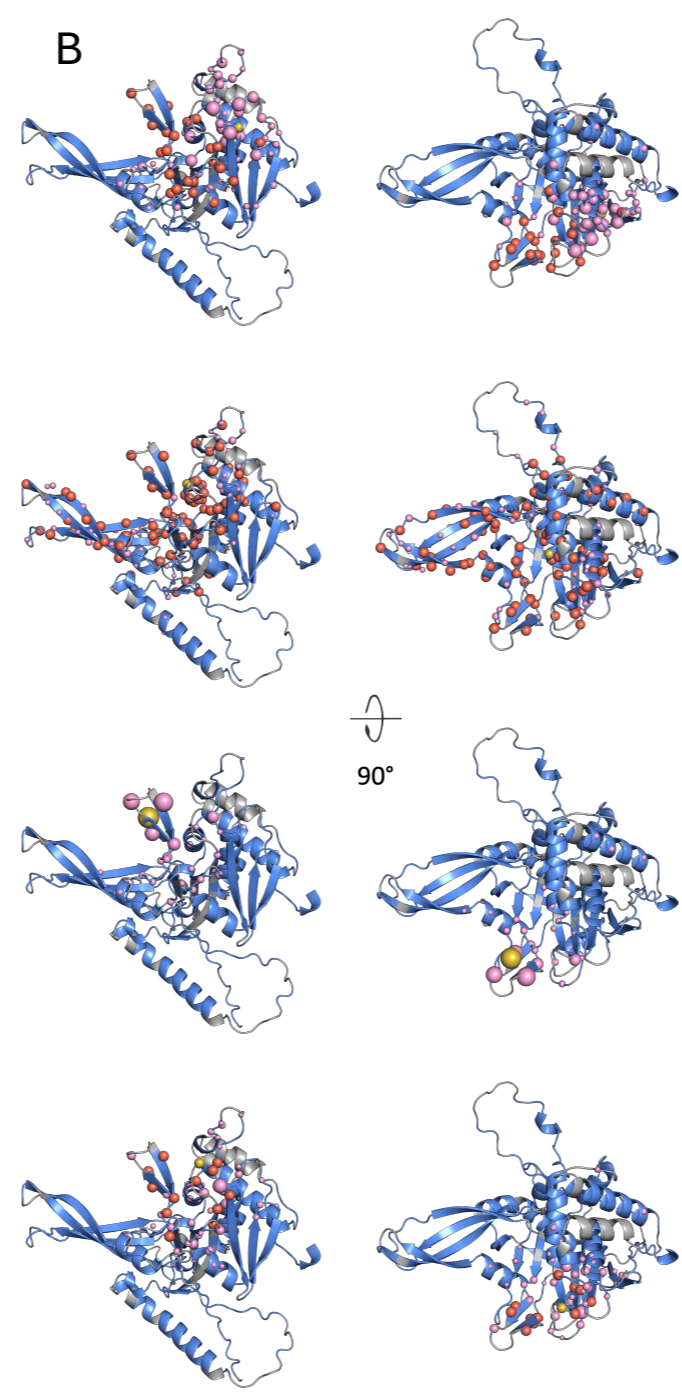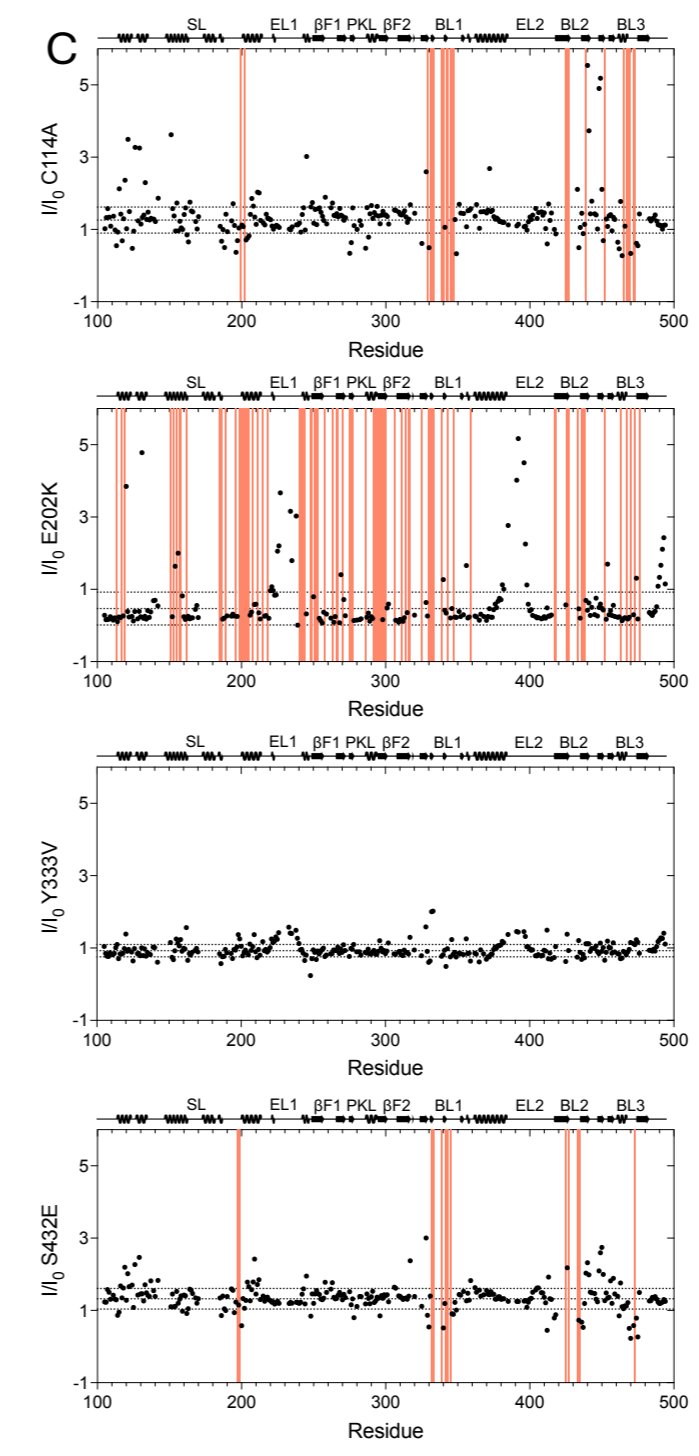

Supplement: Supplementary file 5 — Figure S5. Chemical shift and intensity perturbations of USP14 designed variants. (A) Chemical shift perturbations comparing USP14‐WT and USP14‐C114A, ‐E202K, ‐Y333V and ‐S432E plotted as a function of sequence. Red lines show missing peaks or peaks that have moved so much that assignment was not possible indicating large perturbations. (B) CSPs (pink) and missing peaks (red) from (A) are plotted on AlphaFold model. (C) Intensity ratios normalized by concentration USP14‐WT and USP14‐C114A, ‐E202K, ‐Y333V and ‐S432E. Red (see A). [file PRO-34-e70077-s003.pdf]

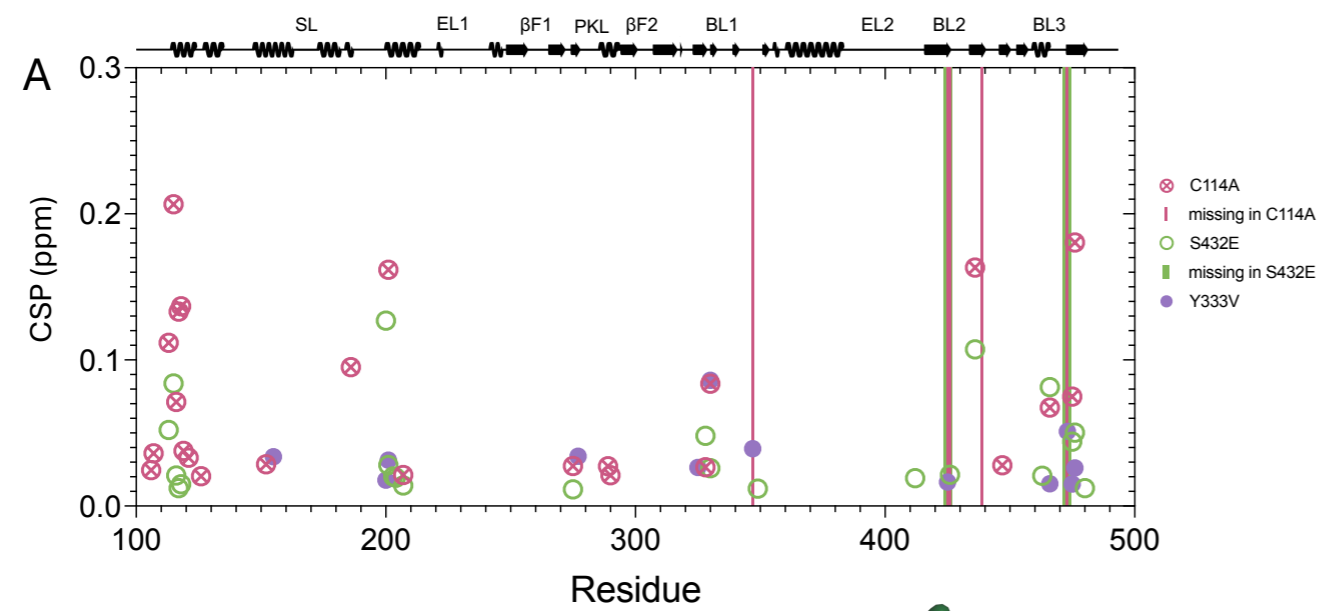

**B**

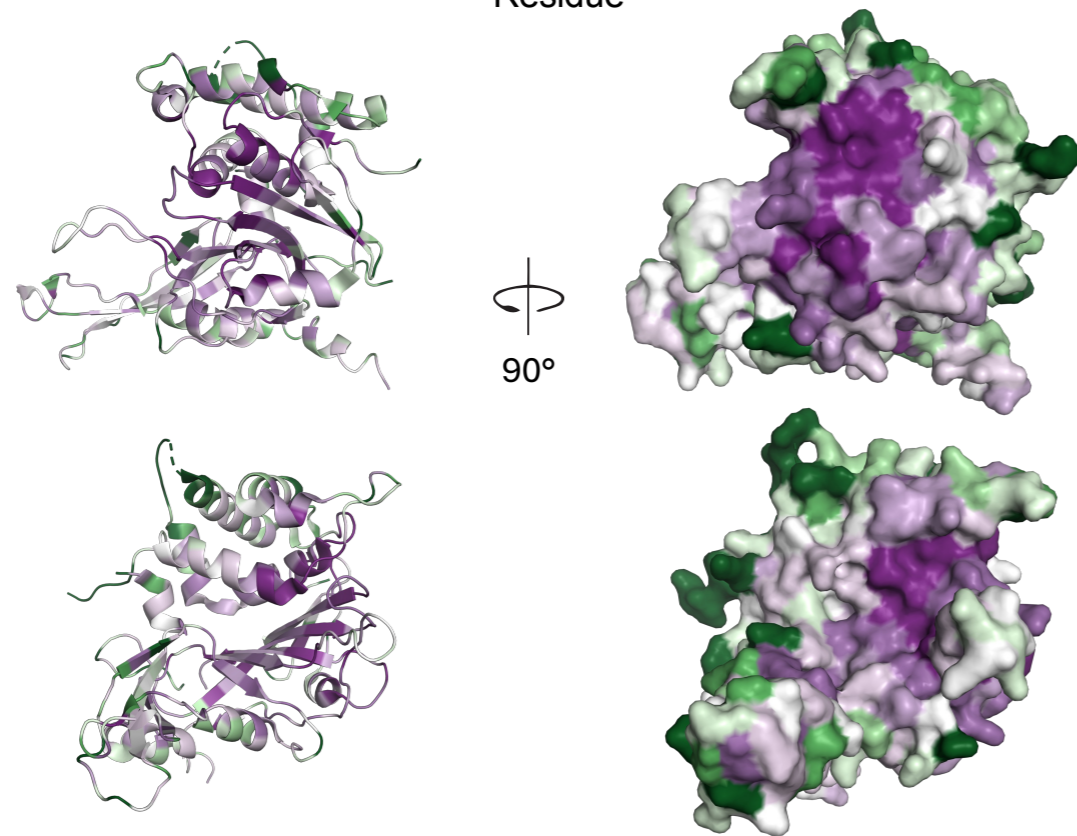

Supplement: Supplementary file 6 — Figure S6. Allosteric networks coincide with conservation. (A) CSPs between USP14‐WT and USP14‐C114A (magenta), ‐S432E (green) and ‐Y333V (purple) of residues that have a summed all‐atom relative solvent accessibility <10 determined by Naccess in the USP14 AlphaFold model. (B) Consurf DB (consurf.tau.ac.il) visualizations of residue conservation mapped onto the USP14USP crystal structure (PDBID: 2AYN, same result for 2AYO). The color scale goes from deep purple (highly conserved) over white to dark green (not conserved). [file PRO-34-e70077-s004.pdf]
